# Toward a Standardized and Individualized Laboratory-Based Protocol for Wheelchair-Specific Exercise Capacity Testing in Wheelchair Athletes: A Scoping Review

**DOI:** 10.1097/PHM.0000000000001941

**Published:** 2021-12-21

**Authors:** Rowie J. F. Janssen, Sonja de Groot, Lucas H. V. Van der Woude, Han Houdijk, Riemer J. K. Vegter

**Affiliations:** From the Center for Human Movement Sciences, University of Groningen, University Medical Center Groningen, Groningen, the Netherlands (RJFJ, LHVVdW, HH, RJKV); Amsterdam Rehabilitation Research Center Reade, Amsterdam, the Netherlands (SdG); Department of Human Movement Sciences, Faculty of Behavioural and Movement Sciences, VU University, Amsterdam, the Netherlands (SdG); Center for Rehabilitation, University Medical Center Groningen, Groningen, the Netherlands (LHVVdW); and Peter Harrison Centre for Disability Sports, School of Sport, Exercise and Health Sciences, Loughborough University, Loughborough, United Kingdom (LHVVdW, RJKV).

**Keywords:** Paralympic, Wheelchair Sport, Performance, Exercise Tests

## Abstract

Previous studies on handrim wheelchair–specific (an)aerobic exercise capacity in wheelchair athletes have used a diversity of participants, equipment, and protocols. Therefore, test results are difficult to compare among studies. The first aim of this scoping review is to provide an overview of the populations studied, the equipment and protocols used, and the reported outcomes from all laboratory-based studies on wheelchair-specific exercise capacity in wheelchair athletes. The second aim is to synthesize these findings into a standardized, yet individualized protocol. A scoping literature search resulted in 10 anaerobic and 38 aerobic protocols. A large variety in equipment, protocol design, and reported outcomes was found. Studies that systematically investigated the influence of protocol features are lacking, which makes it difficult to interpret and compare test outcomes among the heterogeneous group of wheelchair athletes. Protocol design was often dependent on a priori participant knowledge. However, specific guidelines for individualization were missing. However, the common protocol features of the different studies were united into guidelines that could be followed when performing standardized and individualized wheelchair-specific exercise capacity tests in wheelchair athletes. Together with guidelines regarding reporting of participant characteristics, used equipment, and outcome measures, we hope to work toward more international agreement in future testing.

Paralympic wheelchair athletes continuously try to improve their performance and use performance testing to monitor their performance. In the current format of the Paralympic games, five types of handrim wheelchair sports are present in which wheelchair mobility performance, that is, the wheelchair-athlete ability on court/track, is thought to be critical: wheelchair basketball, rugby, tennis (wheelchair court sports), athletics, and triathlon (wheelchair racing) (Paralympic.org). These wheelchair sports are performed by relatively small groups of athletes with a plethora of different disabilities due to trauma or disease, which results in high variability of physical performance.^1^ Moreover, various types of handrim wheelchairs are designed for the specific sport disciplinary demands and conditions of use in each sport.^2^ For instance, compared with wheelchair court sports, athletes competing in wheelchair racing are using a more horizontally positioned wheelchair, which has a lower seat and longer frame, has three larger wheels, a smaller hand rim, and limited steering ability.^3–5^

The current scoping review focusses on the laboratory-based part of performance testing. A laboratory-based environment allows researchers and practitioners to impose controlled workloads and objectively measure the power output that is produced by an individual. Ideally these outcomes are used complementary to the results of field-based tests, because the strength of one approach is the weakness of the other. For example, during field-based testing, the athlete can be tested in their natural environment that results in a higher external validity, yet the changing environmental conditions make standardization difficult.^6^ A conceptual framework that shows the combined strength of laboratory and field-based testing is presented in Figure 1. Because the physical and wheeling performance should be evaluated in concert, the preferred mode for exercise capacity testing should involve task-specific wheelchair propulsion. By using a (wide) treadmill or wheelchair ergometer, the wheelchair-user combination can remain unaltered.^7^

**FIGURE 1:**
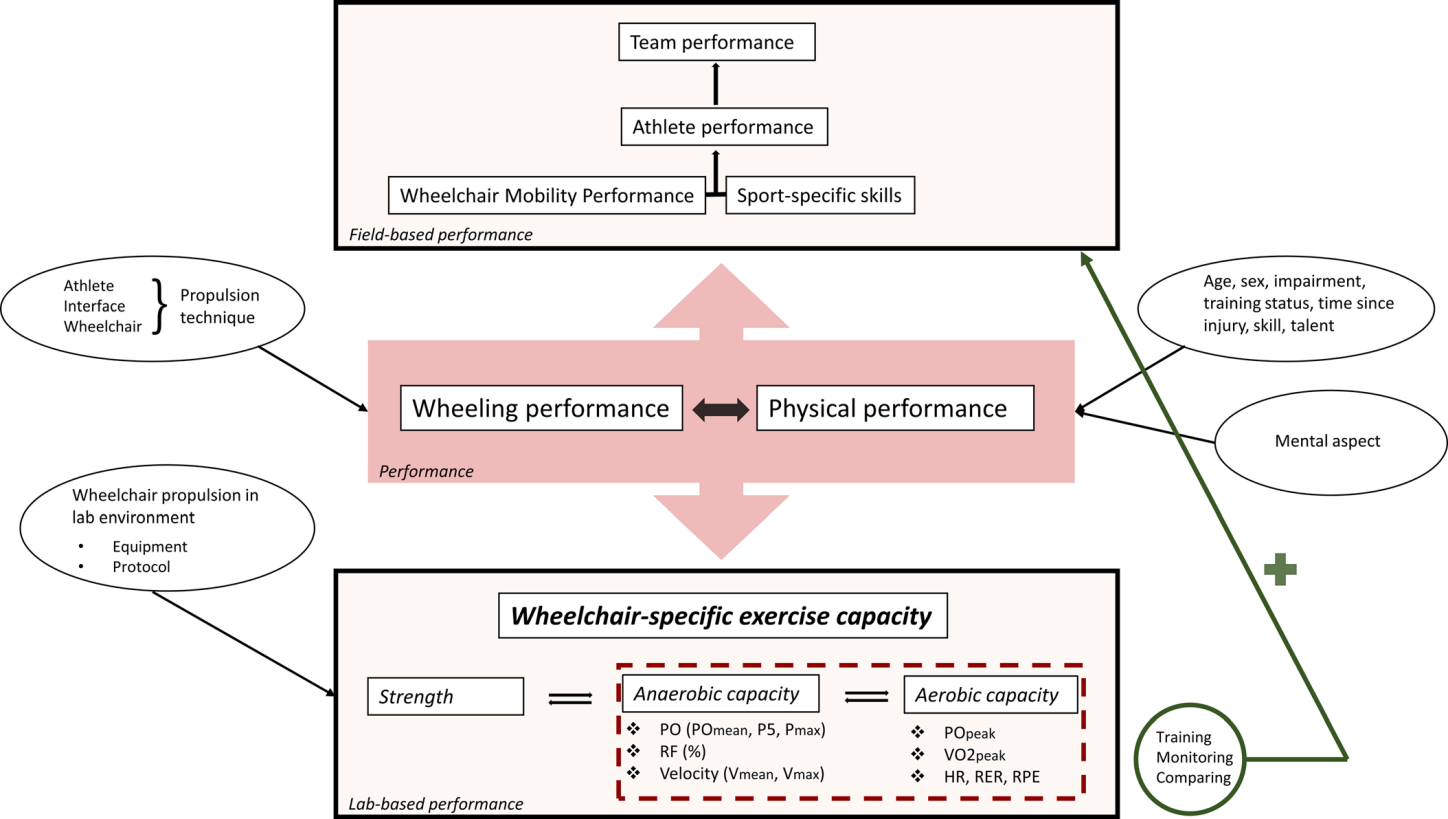
Conceptual framework of wheelchair-specific exercise capacity testing in the context of the wheelchair athlete’s (and team’s) performance. The performance consists most importantly of the wheeling and the physical performance and is influenced by many factors. These aspects can be measured together in the field as the wheelchair mobility performance, and together with the sport-specific skills, it determines the athlete (and team) performance. In the laboratory, they can be measured as wheelchair-specific exercise capacity, which can be divided in three main components: strength, anaerobic capacity, and aerobic capacity. The capacity should be assessed to eventually improve training strategies and monitor and compare athletes, which subsequently helps them perform better in the field. This review focusses on the measurement of anaerobic and aerobic capacity in the laboratory-based environment.

Both the wheelchair-specific anaerobic capacity and aerobic capacity are important for the performance in wheelchair sport activities.^1,8^ The specific demands of each sports discipline determines the relative importance of each of these exercise capacity dimensions. The anaerobic capacity seems more important during the 100–200 m track sprints or for repeated sprints in wheelchair court sports, whereas the aerobic capacity is suggested to be more relevant in maintaining high speeds over a longer duration.

The anaerobic capacity is important for the short-term performance, up to 1–2 min and is most often estimated with the power generated during a sprint or Wingate test.^8^ During a sprint test, the rolling resistance (on a wheelchair ergometer) is set close to the rolling resistance experienced on a sport-specific surface and can be set similar for every individual within a study.^9^ A Wingate test is usually performed at a higher resistance to lower the attained velocity and to potentially reduce hand speed–related coordination problems that may impact the attainable anaerobic power.^10^ In this case, the proper resistance should be set individually, that is, if the set resistance is too high, the athlete is not able to accelerate the wheelchair, whereas a low resistance will lead to coordination problems. Sprint or Wingate tests both aim to examine the explosive sprint and acceleration capabilities of the athletes, as well as the ability to maintain the sprint for a certain duration.^11^ Choices in protocol design (e.g., resistance, duration) will have an influence on the outcomes, as already seen in a study with varying resistances on a wheelchair ergometer^10^ and in the Wingate test on a bicycle ergometer.^12^

The aerobic capacity reflects the cardiorespiratory fitness, which is the ability to perform dynamic, moderate-to-vigorous intensity exercise with the largest possible muscle mass for prolonged periods.^11^ Peak oxygen uptake (VO_2peak_) and peak aerobic power output have been identified as important descriptors of the aerobic capacity and can be assessed with a graded exercise test (GXT).^13–17^ It has been advised to aim for a test duration between 8 and 12 min to reach maximal cardiorespiratory capacity.^18^ Shorter protocols tend to induce muscle fatigue, and longer protocols tend to induce a higher body temperature, dehydration, discomfort, or ventilatory muscle fatigue.^18^ To achieve this, increments of the GXT should be individually tuned. Besides this, and similar to the anaerobic testing modality, multiple protocol designs (e.g., initial load, duration increments) can be used, which again affects the outcomes.^19^

Outcomes of standardized wheelchair-specific (an)aerobic exercise capacity tests not only provide indicators for individual peak exercise capacity but also can be used to individualize training programs, monitor the progress of the athlete over the season, and allow comparison among athletes (Fig. 1). To that end, the use of the same individualized protocol under the same standardized conditions, within and among athletes, is essential.^10,12,19^

Previous research and practice have used a diversity of custom-built treadmills and ergometers and a variety of protocol designs to attain the wheelchair-specific (an)aerobic exercise capacity of wheelchair athletes in a standardized laboratory environment.^20,21^ All tests were developed with a common aim, yet because of their diversity in included participants, equipment, and protocols, which all have an influence on the attained results, test results are difficult to compare among studies. The first aim of this scoping review is to provide a detailed overview of the populations studied, the equipment and protocols used, and the reported outcomes from all studies that addressed the wheelchair-specific anaerobic and/or aerobic exercise capacity in wheelchair athletes. Based on this scoping literature overview, the second aim is to synthesize these different tests into a standardized, yet individualized protocol to assess the wheelchair-specific exercise capacity in athletes competing in different handrim wheelchair sport disciplines.

## MATERIALS AND METHODS

The scoping review was conducted according to previously developed guidelines.^22,23^ The selection process of identification, screening, eligibility, and inclusion was performed in accordance to the Preferred Reporting Items for Systematic Reviews and Meta-Analyses guidelines for scoping reviews (Supplementary Appendix 1, Supplemental Digital Content 1, http://links.lww.com/PHM/B449).^24^

### Search Strategy

For this scoping review, a literature search was conducted in the databases of PubMed, Web of Science, Embase and CINAHL. No date limit was chosen and the following search terms were used on 2021-02-15: (Wheelchair*) AND (Ergomet* OR Dynamomet* OR Simulator OR Treadmill) AND (Sport OR Athlet* OR Para-Athlet*) AND (Exercise test* OR Laboratory based test* OR Maximal incremental test* OR Multistage test* OR Aerobic test* OR Sprint test* OR Wingate OR Anaerobic test*).

### Eligibility Screening and Study Selection

Studies were first selected on basis of title and abstract (RJFJ). For inclusion, a study had to specify the following items: (1) protocol involved wheelchair propulsion in a laboratory environment, (2) protocol description was included, and (3) outcome parameters included (peak) power output for anaerobic tests and either peak oxygen uptake or peak power output for aerobic tests. Exclusion criteria were: field tests, arm crank ergometry, below national level athletes, no quantitative data provided, and other languages than English. Full text was obtained if the abstract met the inclusion and exclusion criteria or when there was not enough information available in the abstract to exclude it. When shown in the full-text that a study did not comply with the previous criteria, it was excluded. Of the studies included in the review, reference lists were scanned for other relevant studies that may have been missed in the search. When two articles were published from the same wheelchair-specific exercise capacity test but focused on another part of the results, only one of those studies was included or the information was merged, accounting for one test.

### Data Extraction

From the included studies, participant’s characteristics, equipment, protocol design, and test outcomes were extracted. Participant’s characteristics reports the study group (i.e., sample size, sex, designated subgroups), practiced sport, age (in years), competition level, disability, time since injury (TSI in years), sport experience (in years), and training hours (TH in weeks). Equipment reports the testing device with corresponding sample frequency (in hertz) and used wheelchair. Protocol design characteristics for the anaerobic tests are the duration (in seconds), start technique, and imposed resistance. Protocol design characteristics for the aerobic tests are the initial load, increments (both in m/sec, in percentage incline, or other), step time, and total test duration (both in min). Reasoning by the researchers for test protocol choices was additionally extracted. The outcomes of interest for the anaerobic tests are the mean, maximal, and highest 5-sec power output (PO_mean_, PO_max_, P5 in watts), the rate of fatigue (RF in percentages), and the mean and maximal velocity (*V*_mean_, *V*_max_ in m/sec). In addition, other power-related outcomes were extracted. The outcomes of interest for the aerobic tests are the peak power output (PO_peak_ in watts), oxygen uptake (VO_2peak_ in liters per minute and/or in milliliters per minute per kilogram), heart rate (HR_peak_ in beats per minute), respiratory exchange ratio (RER_peak_), and the rate of perceived exertion (RPE). When power-related outcomes were reported unilateral or averaged between sides, they were multiplied with two for the total power output.

## RESULTS

**FIGURE 2:**
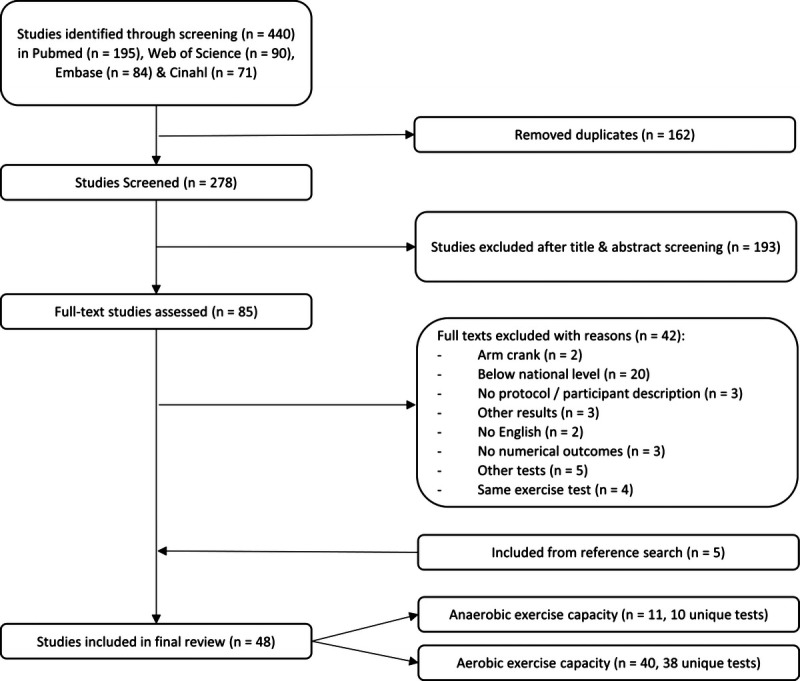
Flow chart of the literature selection process.

The scoping search resulted in 440 studies (Fig. 2). After removing the duplicates, 278 studies remained. Eighty-five studies remained after the initial screening process on title and abstract. Full-text screening excluded 42 studies. Reference list scanning resulted in five additional articles. Thus, a total of 48 studies were included. Eleven studies included an anaerobic test with 10 unique tests, and 40 studies included an aerobic test with 38 unique tests. Three studies included both tests. Supplemental Tables 1 and 2 (Supplemental Digital Content 2, http://links.lww.com/PHM/B450) summarizes the participant’s characteristics, equipment, protocol design, and outcomes for the wheelchair-specific anaerobic and aerobic exercise capacity testing studies, respectively. Both tables are ordered by type of protocol. Table 1 (i.e., anaerobic) is further ordered by duration and start technique; Table 2 (i.e., aerobic) is furthered ordered by step time and total test duration.

### Participant Characteristics

The study sample size, sex, sport, age, competition level, and disability were often well reported. On the contrary, TSI, experience in sport, and TH were only reported by approximately half of the studies (Supplemental Tables 1 and 2, Supplemental Digital Content 2, http://links.lww.com/PHM/B450). Sample size ranged from 1 to 25 athletes.^25,26^ Most studies included only male athletes, nine studies included both sexes, and two studies included only female athletes (Supplemental Tables 1 and 2, Supplemental Digital Content 2, http://links.lww.com/PHM/B450). Athletes active in wheelchair basketball were the most often studied and wheelchair tennis players the least (Supplemental Tables 1 and 2, Supplemental Digital Content 2, http://links.lww.com/PHM/B450). The average age ranged from 23 (6) to 40 (4) years.^27,28^ Most studies included athletes from a national or international level, and three studies only mentioned that their athletes were highly trained.^29–32^ Average TSI ranged from 7 (4) to 23 (13) years,^33,34^ experience in sport from 4 (2) to 15 (8) years,^34,35^ and TH from 5 (2) to 16 (2) hours per week.^29,36^ Nineteen studies defined subgroups in their study population based on sport classification,^26,29,35,37–46^ practiced sport,^1,28,37^ or disability.^36,47–51^

### Equipment

Three different types of equipment were used for testing. Twenty-six studies used a treadmill, 15 studies a roller ergometer, and 4 studies an integrated ergometer. Treadmills are less suited for acceleration and were only used in aerobic tests.^21^ Incremental workloads on a treadmill can be imposed by controlling the slope, velocity, or adding weights via a pulley system to the wheelchair-athlete combination.^52^ Power output can be determined from rolling resistance and impeding gravitational or pulley force and has to be individually conducted through a separate drag test.^53^ The workload of both types of ergometers is controlled by varying either the requested velocity or the imposed resistance, and power output is obtained by multiplying these two quantities.^21^

Treadmills and roller ergometers allow the use of any wheelchair, whereas an integrated ergometer is accommodated with a seat and hand rims that can be individually adjusted.^54^ The majority of the studies, 26 studies, allowed the participants to use their own competition wheelchair (Supplemental Tables 1 and 2, Supplemental Digital Content 2, http://links.lww.com/PHM/B450). In other studies, researchers provided a competition wheelchair,^29,55,56^ they used their own daily life wheelchair,^28,35^ or a combination of the athlete’s competition and daily wheelchair was used (the treadmill was not suitable for racing or wider wheelchairs).^1,16^ Some studies reported that the athlete’s own wheelchair was used, but it remained unclear whether it was a competition or daily life wheelchair.^31,41,57–59^ Three studies did not report on the used wheelchair.^32,60,61^ The four studies that used an integrated ergometer adjusted the wheelchair 3 times to a standardized daily life wheelchair^39,46,62^ and once to the athlete’s competition wheelchair.^63^

### Protocol Design

#### Wheelchair-Specific Anaerobic Exercise Capacity

Six unique sprint (Supplemental Table 1A, Supplemental Digital Content 2, http://links.lww.com/PHM/B450^38,55,56,63–65^) and four Wingate (Supplemental Table 1B, Supplemental Digital Content 2, http://links.lww.com/PHM/B450^39,61,62,66^) protocols were found. Studies that adopted a sprint protocol set a resistance to achieve sport-specific velocities. Wingate protocols set a resistance, so that the maximal velocity stayed less than 3 m/sec^39,61,62^ or 100 wheel rotations per minute.^66^

The duration of the sprint protocols was sport specific and varied from 5 to 30 sec, whereas all Wingate protocols had a duration of 30 sec, chosen in line with the original definition of the anaerobic Wingate protocol on a bicycle ergometer.^67^ Start technique was either from a stationary start or as a flying start. In a stationary start, athletes started from 0 m/sec, which was done for standardization purposes.^38,39,61,62^ In a flying start, athletes were already in motion before the actual start of the test, which was adopted to exclude the initial acceleration phase at the start,^55,56,65,66^ to make it more sport specific,^63,64^ that is, athletes in some wheelchair court sports are almost always in motion, or the reason was not provided.^66^ Flying starts also differed among themselves, that is, the first 3 sec of the tests were excluded,^55,56^ the test started at a fixed velocity^63,64^ or at 75% of the participant’s maximum velocity.^66^

#### Wheelchair-Specific Aerobic Exercise Capacity

Seven different types of protocols were identified based on the initial load and increments, given in either speed, resistance, slope, power, push frequency, or a combination of these parameters. Twenty-three studies used a protocol with initial load expressed in velocity (Supplemental Tables 2A–D, Supplemental Digital Content 2, http://links.lww.com/PHM/B450). These studies can be subdivided in four groups based on type of increment: eight studies used velocity increments (Supplemental Table 2A, Supplemental Digital Content 2, http://links.lww.com/PHM/B450^25,28,35,40–42,64,68^), four slope (Supplemental Table 2B, Supplemental Digital Content 2, http://links.lww.com/PHM/B450^33,43,59,69^), five velocity and slope (Supplemental Table 2C, Supplemental Digital Content 2, http://links.lww.com/PHM/B450^31,32,34,44,45^), and six resistance (Supplemental Table 2D, Supplemental Digital Content 2, http://links.lww.com/PHM/B450^26,29,46,60,70,71^). Twelve studies used a protocol with initial load expressed in velocity and a slope (Supplemental Tables 2E–G, Supplemental Digital Content 2, http://links.lww.com/PHM/B450). From these 12 studies, six studies made increments with velocity (Supplemental Table 2E, Supplemental Digital Content 2, http://links.lww.com/PHM/B450^30,47,57,58,72,73^), five with slope (Supplemental Table 2F, Supplemental Digital Content 2, http://links.lww.com/PHM/B450^16,36,49–51^), and one with velocity and slope (Supplemental Table 2G, Supplemental Digital Content 2, http://links.lww.com/PHM/B450^1^). Two protocols expressed both their initial load and increments in push frequency and resistance (Supplemental Table 2H, Supplemental Digital Content 2, http://links.lww.com/PHM/B450^37,66^). The initial load and/or increments were individually determined in 22 studies and based on (a combination of) a predicted test duration of ±10 min (range = 6–14 min),^25,26,36,41,42,46,47,49–51,61,64,68^ another test on the same day,^1,41,46,49–51,68,69^ previous visits to the laboratory,^25,26,33,42,43,45,47,64^ participant characteristics,^33,46,47,60^ or previous literature.^30,66,73^

The vast majority adopted a continuous protocol, three studies a discontinuous protocol.^29,71,73^ Step time was most often 1 min (18 studies; Supplemental Table 2, Supplemental Digital Content 2, http://links.lww.com/PHM/B450) and ranged between 15 sec^34^ and 4 min.^29^ Test duration was reported in 19 studies and ranged from 6 to just more than 18 min (Supplemental Table 2, Supplemental Digital Content 2, http://links.lww.com/PHM/B450).

### Outcomes

#### Wheelchair-Specific Anaerobic Exercise Capacity

The PO_mean_ was reported in eight studies and was calculated over the entire duration of the test (Supplemental Table 1, Supplemental Digital Content 2, http://links.lww.com/PHM/B450). The PO_max_ was reported in five studies and calculated in different ways. Four studies defined it as a one sample peak value^62–65^ and one study defined it as a 1-sec maximum.^64^ The P5 was reported 4 times as the highest mean power over a successive 5-sec interval. The RF was reported twice, where different definitions were used: (P5_start_ – P5_end_) / P5_start_ * 100% and (P5_max_ – P5_min_) / P5_max_ * 100%.^39,65^ The *V*_mean_ and *V*_max_ were reported in six and three studies, respectively. Some studies reported extra power-related outcomes, for example, PO_mean_/push or peak power after three cycles.^38,61–63^

#### Wheelchair-Specific Aerobic Exercise Capacity

The PO_peak_ was reported in nine studies and was defined as the average power output over the final stage of that protocol, which varied from 30 sec to 4 min in studies that included the PO_peak_ (Supplemental Table 2, Supplemental Digital Content 2, http://links.lww.com/PHM/B450). The VO_2peak_ was reported in all but one study,^58^ 30 times as an absolute value (in liters per minute) and 29 times relative to body mass (in milliliters per minute per kilogram). Twenty-two studies reported both. It was defined as the average over a range from 10 to 60 sec. The HR_peak_ was reported in 32 studies, which varied from the highest one sample peak value to a 60-sec average. The RER_peak_ was reported in 17 studies with a similar time interval as the VO_2_peak. The RPE was reported in four studies: Borg's 6–20 RPE scale was used 3 times,^30,36,47^ the Category Ratio 10 once.^69^

## DISCUSSION

The aim of this review was to scope the literature on the different protocols used for laboratory-based wheelchair-specific anaerobic and aerobic exercise capacity testing in handrim wheelchair athletes. As expected, wheelchair athletes included in these studies form a heterogeneous group in terms of age, sport, disability, TSI, etc. In addition, testing of this group was conducted with a large variety in equipment and protocol design that inherently may influence the outcomes. Besides, the reported outcomes of the tests were not identical among studies, even if they reported the same variable name. The heterogeneity of the test population, the diversity in equipment, the variety in used test protocols, and the different reported outcomes hamper comparison of study material and drawing conclusions about the influence of specific protocol characteristics. To improve comparison and interpretation in future research, awareness among researchers needs to be improved on the smaller and larger effects of all those sources of variation among studies presented. Effects of variability of participant characteristics, equipment, protocol design, and outcome definitions on the actual comparability of the outcomes will therefore be critically discussed hereinafter. Subsequently, the second aim was to synthesize the reviewed literature into a standardized, yet individualized protocol for future use in handrim wheelchair athletes. The suggested test protocol and reporting guidelines for future research, with a fictional example in the Supplementary Appendix 2 (Supplemental Digital Content 3, http://links.lww.com/PHM/B451), will be presented.

### Participant Characteristics

Participant characteristics were not consistently reported among the included studies. TSI, experience in sport, and TH were missing in half of the studies. To get a total overview of the studied population and to be able to compare with other studies, these characteristics should be mentioned.^39,46,74^ We strongly advise to report all relevant participant characteristics in future studies: sex, age, body mass, height, sport, competition level, impairment, classification, TSI, sport experience, and TH per week. They affect peak performance outcome measures and simply help interpretation of the presented data.

### Equipment

The specifications and measurement modalities differ between treadmills, integrated, and roller ergometers but also within one kind of device differences are present, which complicates comparison of study outcomes between apparatus.^21^ Treadmills offer realistic overground wheelchair propulsion in terms of friction, inertia, and coasting behavior, requiring steering but do not allow anaerobic testing.^75^ In ergometers, steering is not essential, but “on-screen” feedback can be a critical task characteristic.^76^ Because the majority of the included studies used a treadmill, this may explain why fewer studies focused on anaerobic capacity, compared with aerobic capacity (11 vs. 40 studies). Both capacities are, at least to some extent, important in wheelchair court and racing sports and can both be measured on an integrated or roller ergometer.

The majority of the included studies tested the athletes in their own competition wheelchair, which increases the sport-specific validity of the test.^77^ This notion is stressed in studies that evaluated small design changes (e.g., seat height or rim diameter) in the wheelchair-user interface on their effects in metabolic cost, mechanical efficiency, and propulsion technique.^78–80^ Any changes in the individualized sports wheelchair may affect peak performance outcomes. Because wheelchair athletes have highly individualized wheelchairs, we advise to do performance tests in their own competition wheelchair and thus on a wheelchair roller ergometer. To better understand results over time, it is highly recommended to report the wheelchair settings in as much detail as possible at every test moment (e.g., seat positioning, rear wheel camber, wheel/hand-rim size, mass).^77^

### Protocol Design

#### Wheelchair-Specific Anaerobic Exercise Capacity

Sprint and Wingate protocols differ in the set resistance and the reached mean velocity. Resistance was generally lower and mean velocity higher in sprint tests. The power output outcomes (PO_mean_, PO_max_, P5) for the Wingate protocols seemed to be lower in comparison with the sprint protocols, which is the opposite of the reasoning provided by the studies that used a Wingate protocol.^39,61,62,66^ Because of the lower velocity, as a consequence of the higher test resistance, the athlete would not be exposed to coordination problems of the upper body and potentially would reach higher anaerobic power output values.^10^ This contradictory result has a few potential explanations. Studies included different groups of athletes, used varying equipment with several protocols and different outcome measures were reported. For example, the highest PO_mean_ and PO_max_ were found in a 5-sec sprint protocol, with a flying start and with a tennis racket, that included wheelchair tennis athletes.^63^ The lowest PO_mean_ and P5 were found in a 30-sec Wingate protocol, from stationary start, that included wheelchair track athletes from the lowest classification group.^39^ P5 was not reported in the first study, PO_max_ not in the second. The same fixed wheelchair ergometer was used, while differently adjusted to the participant (daily living vs. individual competition wheelchair). It is impossible to derive the influence of a sprint or Wingate protocol on test outcomes, because of numerous differences in included participants, equipment, protocol design, and reported outcomes. This underlines the need for better standardization.

Conclusions about the most appropriate anaerobic test are thus difficult to draw from this review because of the many differences between studies. If the aim is to measure the anaerobic capacity, rather than to mimic a field-test sprint, we do suggest a Wingate test with an increased resistance to avoid coordination problems of the upper body and reach a higher anaerobic power.^39,61,62,66^ To make sure that the anaerobic energy system is maximally triggered, we opt for a duration of 30 sec.^81^ A stationarity start is preferable over a flying start because it is the most standardized.

Studies that included a Wingate test set a resistance to keep the wheel velocity less than 3 m/sec.^39,61,62^ The resistance was set individually by the researchers based on participant characteristics. However, the exact “how-to” was unclear, which hampers reproducibility. Studies in the field of rehabilitation that performed a Wingate test had the same aim, to stay less than 3 m/sec, but had a more standardized way of calculating the individual resistance.^82–86^ It was calculated based on an estimated power output (through a previous test) and a mean wheel velocity of 2 m/sec. Wingate tests in this review have been performed in either fixed wheelchairs^39,62,82–86^ or a rugby wheelchair.^61^ Racing wheelchairs have much smaller hand rim diameters, which allows for higher wheel velocities with the same linear hand velocity (at the cost of a higher resistance).^80^ Thus, instead of aiming for a mean wheel velocity of 2 m/sec, we suggest to rather standardize for a mean linear hand velocity of 2 m/sec.^10^

#### Wheelchair-Specific Aerobic Exercise Capacity

The seven identified protocols differ in type of initial load and increment type. Similar to the anaerobic protocol, but with even more studies, comparisons between protocol types are difficult to make because of the heterogeneity of wheelchair athletes, the use of different equipment, different protocol design, and the lack of identical outcome measures.

The majority of the studies made increments in (solely or partly) velocity (21 studies, Supplemental Tables 2A, C, E, Supplemental Digital Content 2, http://links.lww.com/PHM/B450). This results in high propulsion velocities, which may lead to coordination problems of the upper limbs, rather than to their maximal aerobic capacity.^10^ To minimize the influence of possible high-speed coordination problems, a protocol with increments in resisting force is preferred. This can be done either by increasing the slope of a treadmill, by using a pulley system and adding more weight to the pulley on a treadmill, or by increasing the resistance of an ergometer. However, sole increments in slope will results in high slopes and lifting of the front wheels of the wheelchair can occur.^32^ By using increments in resisting force, propulsion velocity remains constant and can be set to a comfortable speed matched to the participant’s ability, also done by multiple studies included in this review.^43,46,49–51,69^

The vast majority adopted a continuous protocol, which is suggested to give similar results as a discontinuous protocol and is less time consuming.^87^ One third of the included studies explained that the magnitude of the initial load and increment were chosen to aim for a certain test duration (range = 6–14 min).^25,26,36,41,42,46,47,49–51,64^

Protocols with a step length of 3 min are often used to reach a steady state in each step, which is sometimes preferred by researchers to determine the submaximal mechanical efficiency and ventilatory thresholds, where the latter can be used for training purposes.^88^ However, a study that investigated the peak physiological responses and the determination of ventilatory thresholds between a 3-min, 1-min, and RAMP protocol, using arm-crank ergometry, showed no differences in the identification of ventilatory thresholds.^19^ In addition, a higher PO_peak_ was found for the shorter protocols.^19^ It needs to be noted that no studies were included in this review that adopted a RAMP protocol with a linear increase in workload. Further research is needed to investigate the utility in wheelchair exercise tests of such a RAMP protocol. For now, a continuous protocol with an aimed duration between 8 and 12 min^18^ and steps of 1 min is recommended.

### Outcomes

For the anaerobic test, we suggest to adhere to the original outcomes of the Wingate test,^67^ which are also the most often used among the included studies. This contains the PO_mean_, PO_max_, and P5 (in watts), which are calculated as the average power output over the total test duration, the one sample highest value, and the highest mean power over successive 5-sec intervals, respectively. The RF as (P5_start_ – P5_end_) / (P5_start_) * 100%. Last, *V*_mean_ and *V*_max_ in m/sec.

For the aerobic test, we suggest to use the VO_2peak_ as a 30-sec average, in liters per minute and in milliliters per minute per kilogram. The PO_peak_ (in watts and in watts per kilogram) of the last completed step plus every 30 sec in the noncompleted step is taken into account by adding for each 30 sec: 1 / (step length / 30 sec) times the PO increment in the noncompeted step.^89^ Secondary criteria are often used to assess the validity of the VO_2peak_ (VO_2plateau_: ≤2 ml/min/kg change over the last two stages, HR_peak_ > 95% of age-predicted maximum and RER_peak_ > 1.10).^90^ The HR_peak_ is defined as the highest peak value and RER_peak_ in the same time interval as the VO_2peak_. Because the HR regulation might be affected in individuals with a SCI > T6, we advise to additionally use an RPE greater than 8 or 18 as a secondary criteria, depending on the scale.^90^ Laboratory tests often adopt the Borg's 6–20 RPE scale, field tests the Category Ratio 10.^91,92^ However, these can be converted to each other and thus interchangeably used.^93^

### Suggested Guidelines for the Standardization of (an)Aerobic Test Protocols

The resistance in the Wingate test and the increments in the GXT needs to be individually determined. Research found in this review did this mainly based on previous visits and/or participant characteristics.^25,36,39,41,42,46,49–51,61,62,64,68^ However, no general guidelines on why and how the specific resistances and increments were chosen were presented, hampering the reproducibility by other researchers.

A number of rehabilitation studies^82–86^ have adopted a standardized, yet individualized protocol that uses the relation between isometric force–anaerobic and aerobic power as a predictor for the most suited resistance.^94^ Isometric force can be measured in a standardized way and performed by each individual with the same settings.^82–85,95^ The participants exerts maximal force for 5 sec on the top of the blocked hand rims (ergometer or alternative set up).^82,83^ The previously mentioned associations are more specifically between the highest 3-sec isometric user force, the mean power over a 30-sec Wingate test (PO_mean_), and the peak power in a GXT (PO_peak_).^94^ Thus, if the isometric force is measured, PO_mean_ can be predicted, and with a mean hand velocity of 2 m/sec, the individual resistance of the Wingate test can be calculated. Similarly, PO_peak_ can be predicted from PO_mean_ and resistance increments can be individually scaled to achieve a duration of 8 to 12 min in the GXT. All calculations are presented in the Supplementary Appendix 2 (Supplemental Digital Content 3, http://links.lww.com/PHM/B451).

### Future Research

To draw conclusions about the influence of protocol characteristics on both the wheelchair-specific anaerobic and aerobic exercise capacity outcomes, aspects of protocol design should be investigated in isolation. The same group of individuals should be tested twice with only one change in the protocol design (e.g., stationary vs. flying start in the anaerobic test). Such studies are currently lacking in the wheeled adapted sports domain.

In addition, the common features of equipment and test protocols discussed in this review were united in a proposed protocol for future standardization, which is summarized in the Supplementary Appendix 2 (Supplemental Digital Content 3, http://links.lww.com/PHM/B451). A roller ergometer allows anaerobic exercise tests and the use of the athlete’s individualized competition wheelchair.^21^ To build the protocol for (an)aerobic exercise tests, associations between the isometric force–anaerobic and aerobic power can be used to predict outcomes, and accordingly, protocols can be individually scaled.^94^ Together with guidelines regarding participant characteristics and test outcomes, it becomes possible to compare among the various groups of wheelchair athletes and allow an easier interpretation of test outcomes.

These associations between the isometric force–anaerobic power and aerobic power were developed among a group of handrim wheelchair–dependent male individuals with a chronic spinal cord injury^94^ and the use of this protocol is still limited to few rehabilitation and experimental studies.^82–85^ Previous research in male wheelchair track athletes showed a similar association between the anaerobic and aerobic capacity what might indicate that these associations are somehow similar in wheelchair athletes.^46^ However, future research should evaluate the usability of this association and protocol in a group of wheelchair athletes. It remains questionable if the previously found associations allow scaling of the test protocols in all individuals or whether they need to be reconsidered for each sport and/or classification.

## CONCLUSIONS

With this scoping review, we provided a highly variable perspective on the athlete population, equipment, test protocols, and reported outcomes of the wheelchair-specific exercise capacity in international literature. This variability in equipment (e.g., daily life wheelchair or sports wheelchair), test protocols (e.g., step time aerobic test), and reported outcomes (e.g., PO_mean_ and/or P5 and/or PO_max_ in the anaerobic test) makes it difficult to interpret the anaerobic and aerobic test outcomes and compare results among the heterogeneous group of wheelchair athletes (e.g., practiced sport, TH/week). Yet, the common features were united into a proposed protocol and reporting guidelines for future standardization. These guidelines should at least be used to report all relevant participant characteristics, equipment specifications, protocol design, and same test outcomes. Preferable, researchers should adopt the standardized, yet individualized protocol for wheelchair-specific anaerobic and aerobic exercise capacity testing. By having a protocol based on common principles, albeit scaled to the individual, athletes will be provided with interathlete and intra-athlete comparable measurements of their handrim wheelchair–specific exercise capacity, to ultimately improve their sport performance.

## Supplementary Material
