# Supplementary material for: Toward a Standardized and Individualized Laboratory-Based Protocol for Wheelchair-Specific Exercise Capacity Testing in Wheelchair Athletes: A Scoping Review
Source: Am J Phys Med Rehabil. 2021 Dec 21;102(3):261–9. doi: 10.1097/PHM.0000000000001941 (PMC9940834; doi:10.1097/PHM.0000000000001941)
Supplement: Supplementary file 2 [file ajpmr-102-261-s002.docx]

| **Testing guidelines**  ***Wheelchair-specific exercise capacity*** | | |
| --- | --- | --- |
|  | | Example (fictive data)  Test date: 09-04-2021 |
| **PARTICIPANT CHARACTERISTICS** | | |
|  | Sex | Female |
|  | Age (yrs) | 26 |
|  | Body Mass (kg) | 69 |
|  | Height (cm) | 180 |
|  | Sport | Wheelchair basketball |
|  | Competition level | National |
|  | Impairment | PP |
|  | Classification | 4.0 |
|  | Time since injury (yrs) | 21 |
|  | Sport experience (yrs) | 12 |
|  | Training hours per week | 14 |
|  | Extra reported participant characteristics: *e.g. role in field = attacker.* | |
| **EQUIPMENT** | | |
|  | Device  *Preferable: roller ergometer that measures power* | LODE Esseda ergometer^80^ |
|  | Sampling rate [Hz] | 100 Hz |
|  | Wheelchair (seat positioning, rear wheel camber, wheel/hand-rim size, mass and tire pressure)  *Preferable: athlete’s sport wheelchair* | Athlete’s sports wheelchair (seat height-front: 0.52m, seat height-back: 0.49m, camber: 48º, wheel size: 0.34m, rim size: 0.31m, mass: 9.5kg, tire pressure: 7 bar) |
|  | Notes: *-* | |
| **ISOMETRIC FORCE** | | |
| **Protocol** | Hand position | Top dead center of the blocked rims |
|  | Duration | 5 s |
|  | Total attempts + rest period | 3 attempts, 2 min rest |
|  | Notes: *-* | |
| **Outcomes** | Fiso [N]  *3s maximal user force of best try, averaged over left and right* | 266 N |
| **ANAEROBIC CAPACITY** | | |
| **Protocol** | Initial load  *Preferable: stationary start* | Stationary start |
|  | Resistance  *Preferable: based on the wheelchair-specific isometric force and the individual linear mean velocity (adapted for hand-rim) which is aimed for to prevent coordination problems.*  *POmean [W/kg] = 0.51 Fiso [N/kg] – 0.18, R = 0.75*  *POmean [W] = POmean * mass user [kg]*  *Vmean [m/s] = (2 [m/s] * radius wheelsize [m])) / radius handrim [m]*  *F_r_ [N] = POmean / Vmean* | Fiso = 266 N ( = 3.9 N/kg)  POmean = 0.51 * 3.9 – 0.18 = 1.81 W/kg  POmean = 1.81 * 69 = 124.8 W  Vmean = (2 * 0.340) / 0.305 = 2.2 m/s  Resistance (F_r_) = 124.8 / 2.2 = 56 N |
|  | Duration  *Preferable: 30 s* | 30 s |
|  | Notes: *-* | |
| **Outcomes** | POmean [W]  *Mean power output over whole test duration, sum of left and right* | 117 W |
|  | P5 [W]  *Highest mean power over successive 5-s intervals, sum of left and right* | 165 W |
|  | POmax [W]  *Maximal power as one sample peak, sum of left and right* | 173 W |
|  | RF [%]  *((P5start - P5end) / P5start ) * 100%* | 41 % |
|  | Vmean [m/s]  *Mean velocity over whole test duration* | 1.9 m/s |
|  | Vmax [m/s]  *Maximal velocity as one sample peak* | 2.6 m/s |
|  | Extra calculated parameters: *e.g. power output over the first three cycles* | |
| **AEROBIC CAPACITY** | | |
| **Protocol** | Increment type  *Preferable: constant velocity with increments in resistance, thus power* | Constant comfortable velocity (3.0 m/s) with increments in resistance |
|  | Initial load & increment size  *Preferable: Individual for each athlete, based on the estimation from the anaerobic exercise test (or isometric force) and the total duration.*  *POaer [W/kg] = 0.67 * POmean [W/kg] + 0.11, R = 0.81*  *(POaer [W/kg]= 0.34 * Fiso [N/kg] – 0.02, R = 0.66)*  *POend [W] = POaer [W/kg] x mass user [kg]*  *POstart [W] = 0.30 * POend*  *POstep [W] = 0.11 * (POend – POstart) ~ if steps are 1 min* | POmean = 117 ( = 1.7 W/kg; previous test)  POaer = 0.67 * 1.7 + 0.11 = 1.25 W/kg  POend = 1.25 * 69 = 86.0 W  POstart = 0.30 * 86.0 = 25.8 W  POstep = 0.11 * (86.0 – 25.8) = 6.6 W |
|  | Duration increment  *Preferable: 1 min* | 1 min |
|  | Aim total duration  *Preferable: 10 min* | 10 min |
|  | Notes: *-* | |
| **Outcomes** | Total duration  *Time at termination of test* | 11:30 |
|  | POpeak [W]  *Highest power output, sum of left and right, adjusted for duration & step time*  *POpeak = POpeak_completed step + (1/x) * POstep*  *x = step time / 30 s* | 92.6 + (1/2) * 6.6 W = 95.9 W |
|  | VO_2_peak [l/min & ml/min/kg]  *Peak oxygen uptake over 30 s* | 2.4 l/min  34.8 ml/min/kg |
|  | VO_2_ plateau  *Small increase (≤ 2 ml/min/kg) over the last two stages* | No |
|  | HRpeak [bpm]  *Peak heart rate* | 189 bpm |
|  | RERpeak  *Peak respiratory exchange ratio over 30 s* | 1.21 |
|  | RPE [Borg scale 1-10 or 6-20]  *Rate of perceived exertion at termination test* | 10 [scale: 1-10] |
|  | Extra calculated parameters: *e.g. VE (ventilation rate per min)* | |
