# Supplementary material for: Toward a Standardized and Individualized Laboratory-Based Protocol for Wheelchair-Specific Exercise Capacity Testing in Wheelchair Athletes: A Scoping Review
Source: Am J Phys Med Rehabil. 2021 Dec 21;102(3):261–9. doi: 10.1097/PHM.0000000000001941 (PMC9940834; doi:10.1097/PHM.0000000000001941)
Supplement: Supplementary file 3 [file ajpmr-102-261-s003.docx]

**Towards a standardized and individualized lab-based protocol for wheelchair-specific exercise capacity testing in wheelchair athletes: a scoping review**

**Rowie J.F. Janssen^1*^, Sonja de Groot^2,3^, Lucas H.V. Van der Woude^1,4,5^, Han Houdijk^1^, Riemer J.K. Vegter^1,5^**

*^1^ University of Groningen, University Medical Center Groningen, Center for Human Movement Sciences, The Netherlands; ^2^ Amsterdam Rehabilitation Research Center Reade, Amsterdam, The Netherlands; ^3^ Department of Human Movement Sciences, Faculty of Behavioural and Movement Sciences, VU University, Amsterdam, The Netherlands. ^4^ Center for Rehabilitation, University Medical Center Groningen, Groningen, The Netherlands; ^5^* *Peter Harrison Centre for Disability Sports, School of Sport, Exercise and Health Sciences, Loughborough University.*

*Corresponding author: [r.j.f.janssen@umcg.nl](mailto:r.j.f.janssen@umcg.nl)

**Conflict of Interest statement:** The authors declare that they have no conflict of interest.

**Funding:** This review was funded by ZonMw (Wheelpower, project number: 546003002).

**Towards a standardized and individualized lab-based protocol for wheelchair-specific exercise capacity testing in wheelchair athletes: a scoping review**

Previous studies on handrim wheelchair-specific (an)aerobic exercise capacity in wheelchair athletes have used a diversity of participants, equipment and protocols. Therefore, test results are difficult to compare among studies. The first aim of this scoping review is to provide an overview of the populations studied, the equipment and protocols used and the reported outcomes from all lab-based studies on wheelchair-specific exercise capacity in wheelchair athletes. The second aim is to synthesize these findings into a standardized, yet individualized protocol. A scoping literature search resulted in 10 anaerobic and 38 aerobic protocols. A large variety in equipment, protocol design and reported outcomes was found. Studies that systematically investigated the influence of protocol features are lacking, which makes it difficult to interpret and compare test outcomes among the heterogeneous group of wheelchair athletes. Protocol design was often dependent on a-priori participant knowledge. However, specific guidelines for individualisation were missing. Yet, the common protocol features of the different studies were united into guidelines that could be followed when performing standardised and individualized wheelchair-specific exercise capacity tests in wheelchair athletes. Together with guidelines regarding reporting of participant characteristics, used equipment and outcome measures we hope to work towards more international agreement in future testing.

**Key words:** Paralympic, Wheelchair Sport, Performance, Exercise tests

**Introduction**

Paralympic wheelchair athletes continuously try to improve their performance and use performance testing to monitor their performance. In the current format of the Paralympic games, five types of handrim wheelchair sports are present in which wheelchair mobility performance, i.e. the wheelchair-athlete ability on court/track, is thought to be critical: wheelchair basketball, rugby, tennis (wheelchair court sports), athletics and triathlon (wheelchair racing) (Paralympic.org). These wheelchair sports are performed by relatively small groups of athletes with a plethora of different disabilities due to trauma or disease, which results in high variability of physical performance.^1^ Moreover, various types of handrim wheelchairs are designed for the specific sport disciplinary demands and conditions of use in each sport.^2^ For instance, compared with wheelchair court sports, athletes competing in wheelchair racing are using a more horizontally positioned wheelchair which has a lower seat and longer frame, has three larger wheels, a smaller hand rim and limited steering ability.^3–5^

The current scoping review focusses on the lab-based part of performance testing. A lab-based environment allows researchers and practitioners to impose controlled workloads and objectively measure the power output that is produced by an individual. Ideally these outcomes are used complementary to the results of field-based tests, since the strength of one approach is the weakness of the other. For example, during field-based testing the athlete can be tested in their natural environment which results in a higher external validity, yet the changing environmental conditions make standardisation difficult.^6^ A conceptual framework that shows the combined strength of lab and field-based testing is presented in Figure 1. Because the physical and wheeling performance should be evaluated in concert, the preferred mode for exercise capacity testing should involve task-specific wheelchair propulsion. By using a (wide) treadmill or wheelchair ergometer, the wheelchair-user combination can remain unaltered.^7^

Both the wheelchair-specific anaerobic and aerobic capacity are important for the performance in wheelchair sport activities.^1,8^ The specific demands of each sports discipline determines the relative importance of each of these exercise capacity dimensions. The anaerobic capacity seems more important during the 100-200m track sprints or for repeated sprints in wheelchair court sports, while the aerobic capacity is suggested to be more relevant in maintaining high speeds over a longer duration.

The anaerobic capacity is important for the short-term performance, up to 1-2 minutes, and is most often estimated with the power generated during a sprint or Wingate test.^8^ During a sprint test, the rolling resistance (on a wheelchair ergometer) is set close to the rolling resistance experienced on a sport-specific surface and can be set similar for every individual within a study.^9^ A Wingate test is usually performed at a higher resistance to lower the attained velocity, and to potentially reduce hand-speed related coordination problems that may impact the attainable anaerobic power.^10^ In this case, the proper resistance should be set individually, i.e. if the set resistance is too high, the athlete is not able to accelerate the wheelchair, while a low resistance will lead to coordination problems. Sprint or Wingate tests both aim to examine the explosive sprint- and acceleration capabilities of the athletes, as well as the ability to maintain the sprint for a certain duration.^11^ Choices in protocol design (e.g. resistance, duration) will have an influence on the outcomes, as already seen in a study with varying resistances on a wheelchair ergometer^10^ and in the Wingate test on a bicycle ergometer.^12^

The aerobic capacity reflects the cardiorespiratory fitness, which is the ability to perform dynamic, moderate-to-vigorous intensity exercise with the largest possible muscle mass for prolonged periods of time.^11^ Peak oxygen uptake (VO_2_peak) and peak aerobic power output have been identified as important descriptors of the aerobic capacity and can be assessed with a graded exercise test (GXT).^13–17^ It has been advised to aim for a test duration between 8-12 minutes to reach maximal cardio-respiratory capacity.^19^ Shorter protocols tend to induce muscle fatigue, longer protocols a higher body temperature, dehydration, discomfort or ventilatory muscle fatigue.^19^ In order to achieve this, increments of the GXT should be individually tuned. Besides this, and similar to the anaerobic testing modality, multiple protocol designs (e.g. initial load, duration increments) can be used which again affects the outcomes.^20^

Outcomes of standardized wheelchair-specific (an)aerobic exercise capacity tests not only provide indicators for individual peak exercise capacity, but can also be used to individualize training programs, monitor the progress of the athlete over the season and allows comparison among athletes (Figure 1). To that end, the use of the same individualized protocol under the same standardized conditions, within and among athletes is essential.^10,12,20^

Previous research and practice have used a diversity of custom-built treadmills and ergometers and a variety of protocol designs to attain the wheelchair-specific (an)aerobic exercise capacity of wheelchair athletes in a standardized lab environment.^21,22^ All tests were developed with a common aim, yet because of their diversity in included participants, equipment and protocols which all have an influence on the attained results, test results are difficult to compare among studies. The first aim of this scoping review is to provide a detailed overview of the populations studied, the equipment and protocols used and the reported outcomes from all studies that addressed the wheelchair-specific anaerobic and/or aerobic exercise capacity in wheelchair athletes. Based on this scoping literature overview, the second aim is to synthesize these different tests into a standardized, yet individualized protocol to assess the wheelchair-specific exercise capacity in athletes competing in different handrim wheelchair sport disciplines.

**Materials and Methods**

The scoping review was conducted according to previously developed guidelines.^23,24^ The selection process of identification, screening, eligibility and inclusion was performed in accordance to the Preferred Reporting Items for Systematic Reviews and Meta-Analyses (PRISMA) guidelines for scoping reviews (Supplementary Appendix 1, Supplemental Digital Content 1, http://links.lww.com/PHM/B449).^25^

***Search strategy***

For this scoping review, a literature search was conducted in the databases of PubMed, Web of Science, EMBASE, and CINAHL. No date limit was chosen and the following search terms were used on 2021-02-15: (Wheelchair*) AND (Ergomet* OR Dynamomet* OR Simulator OR Treadmill) AND (Sport OR Athlet* OR Para-Athlet*) AND (Exercise test* OR Laboratory based test* OR Maximal incremental test* OR Multistage test* OR Aerobic test* OR Sprint test* OR Wingate OR Anaerobic test*).

***Eligibility screening and study selection***

Studies were first selected on basis of title and abstract (RJFJ). For inclusion, a study had to specify the following items: (1) protocol involved wheelchair propulsion in a lab environment, (2) protocol description was included, (3) outcome parameters included (peak) power output for anaerobic tests and either peak oxygen uptake or peak power output for aerobic tests. Exclusion criteria were: field tests, arm crank ergometry, below national level athletes, no quantitative data provided, other language than English. Full text was obtained if the abstract met the in- and exclusion criteria or when there was not enough information available in the abstract to exclude it. When shown in the full-text that a study did not comply with the previous criteria it was excluded. Of the studies included in the review, reference lists were scanned for other relevant studies that may have been missed in the search. When two articles were published from the same wheelchair-specific exercise capacity test but focussed on another part of the results, only one of those studies was included or the information was merged, accounting for one test.

***Data Extraction***

From the included studies, participant’s characteristics, equipment, protocol design and test outcomes were extracted. *Participant’s characteristics* reports the study group (i.e. sample size, sex, designated subgroups), practised sport, age [yr], competition level (C-level), disability, time since injury (TSI [yr]), sport experience [yr] and training hours (TH [wk]). *Equipment* reports the testing device with corresponding sample frequency (Fs, [Hz]) and used wheelchair. *Protocol design* characteristics for the anaerobic tests are the duration [s], start technique and imposed resistance. Protocol design characteristics for the aerobic tests are the initial load, increments (both [m/s], [%] or other), step time and total test duration [both min]. Reasoning by the researchers for test protocol choices was additionally extracted. The outcomes of interest for the anaerobic tests are the mean, maximal and highest 5-s power output (POmean, POmax, P5 [W]), the rate of fatigue (RF [%]) and the mean and maximal velocity (Vmean, Vmax [m/s]). Also, other power-related outcomes were extracted. The outcomes of interest for the aerobic tests are the peak power output (POpeak [W]), oxygen uptake (VO_2_peak [l/min and/or ml/min/kg]), heart rate (HRpeak [bpm]), respiratory exchange ratio (RERpeak) and the rate of perceived exertion (RPE). When power-related outcomes were reported unilateral or averaged between sides, they were multiplied with two for the total power output.

**Results**

The scoping search resulted in 440 studies (Figure. 2). After removing the duplicates 278 studies remained. Eighty-five studies remained after the initial screening process on title and abstract. Full text screening excluded 42 studies. Reference list scanning resulted in 5 additional articles. Thus, a total of 48 studies were included. Eleven studies included an anaerobic test with 10 unique tests, 40 studies included an aerobic test with 38 unique tests. Three studies included both tests. Supplemental Table 1 and 2 (Supplemental Digital Content 2, http://links.lww.com/PHM/B450) summarizes the participant’s characteristics, equipment, protocol design and outcomes for the wheelchair-specific anaerobic and aerobic exercise capacity testing studies, respectively. Both tables are ordered by type of protocol. Table 1 (i.e. anaerobic) is further ordered by duration and start technique; Table 2 (i.e. aerobic) by step time and total test duration.

***Participant characteristics***

The study sample size, sex, sport, age, competition level and disability were often well reported. On the contrary, TSI, experience in sport and TH were only reported by approximately half of the studies (Supplemental Tables 1 and 2, Supplemental Digital Content 2, http://links.lww.com/PHM/B450). Sample size ranged from 1 to 25 athletes.^26,27^ The majority of the studies included only male athletes, nine studies included both sexes and two studies included only female athletes (Supplemental Tables 1 and 2, Supplemental Digital Content 2, http://links.lww.com/PHM/B450). Athletes active in wheelchair basketball were the most often studied and wheelchair tennis players the least (Supplemental Tables 1 and 2, Supplemental Digital Content 2, http://links.lww.com/PHM/B450). The average age ranged from 23 (6) to 40 (4) yrs.^28,29^ Most studies included athletes from a national or international level, three studies only mentioned that their athletes were highly-trained.^30–33^ Average TSI ranged from 7 (4) to 23 (13) yrs^34,35^, experience in sport from 4 (2) to 15 (8) yrs^35,36^ and TH from 5 (2) to 16 (2) hrs per week.^30,37^ Nineteen studies defined subgroups in their study population based on sport classification^27,30,36,38–47^, practiced sport^1,29,38^ or disability.^37,48–52^

***Equipment***

Three different types of equipment were used for testing. Twenty-six studies used a treadmill, 15 studies a roller ergometer and four studies an integrated ergometer. Treadmills are less suited for acceleration and were only used in aerobic tests.^22^ Incremental workloads on a treadmill can be imposed by controlling the slope, velocity or by adding weights via a pulley system to the wheelchair-athlete combination.^53^ Power output can be determined from rolling resistance and impeding gravitational or pulley force and has to be individually conducted through a separate drag test.^54^ The workload of both types of ergometers is controlled by either varying the requested velocity or the imposed resistance, power output is obtained by multiplying these two quantities.^22^

Treadmills and roller ergometers allow the use of any wheelchair, while an integrated ergometer is accommodated with a seat and hand rims that can be individually adjusted.^55^ The majority of the studies, 26 studies, allowed the participants to use their own competition wheelchair (Supplemental Tables 1 and 2, Supplemental Digital Content 2, http://links.lww.com/PHM/B450). In other studies, researchers provided a competition wheelchair^30,56,57^, they used their own daily life wheelchair^29,36^ or a combination of the athlete’s competition and daily wheelchair was used (the treadmill was not suitable for racing or wider wheelchairs).^1,16^ Some studies reported that the athlete’s own wheelchair was used, but it remained unclear whether it was a competition or daily life wheelchair.^42,58–61^ Three studies did not report on the used wheelchair.^33,64,65^ The four studies that used an integrated ergometer adjusted the wheelchair three times to a standardized daily life wheelchair^40,47,62^ and once to the athlete’s competition wheelchair.^63^

***Protocol design***

*Wheelchair-specific anaerobic exercise capacity*

Six unique sprint (Supplemental Table 1.A, Supplemental Digital Content 2, http://links.lww.com/PHM/B450^39,56,57,63,66,67^) and four Wingate (Supplemental Table 1.B, Supplemental Digital Content 2, http://links.lww.com/PHM/B450^40,62,65,68^) protocols were found. Studies that adopted a sprint protocol set a resistance to achieve sport-specific velocities. Wingate protocols set a resistance, so that the maximal velocity stayed below 3 m/s^40,62,65^ or 100 wheel rotations per min.^68^

The duration of the sprint protocols was sport-specific and varied from 5 to 30 s, while all Wingate protocols had a duration of 30 s, chosen in line with the original definition of the anaerobic Wingate protocol on a bicycle ergometer.^69^ Start technique was either from a stationary start or as a flying start. In a stationary start, athletes started from 0 m/s, which was done for standardization purposes.^39,40,62,65^ In a flying start, athletes were already in motion before the actual start of the test, which was adopted to exclude the initial acceleration phase at the start^56,57,67,68^, to make it more sport specific^63,66^, i.e., athletes in some wheelchair court sports are almost always in motion, or the reason was not provided.^68^ Flying starts also differed among themselves, i.e., the first three seconds of the tests were excluded^56,57^, the test started at a fixed velocity^63,66^ or at 75% of the participant’s maximum velocity.^68^

*Wheelchair-specific aerobic exercise capacity*

Seven different types of protocols were identified based on the initial load and increments, given in either speed, resistance, slope, power, push frequency or a combination of these parameters. Twenty-three studies used a protocol with initial load expressed in velocity (Supplemental Table 2.ABCD, Supplemental Digital Content 2, http://links.lww.com/PHM/B450). These studies can be subdivided in four groups based on type of increment: eight studies used velocity increments (Supplemental Table 2.A, Supplemental Digital Content 2, http://links.lww.com/PHM/B450^26,29,36,41–43,66,70^), four slope (Supplemental Table 2.B, Supplemental Digital Content 2, http://links.lww.com/PHM/B450^34,44,61,71^), five velocity and slope (Supplemental Table 2.C, Supplemental Digital Content 2, http://links.lww.com/PHM/B450^33,35,45,46,60^) and six resistance (Supplemental Table 2.D, Supplemental Digital Content 2, http://links.lww.com/PHM/B450^27,30,47,64,72,73^). Twelve studies used a protocol with initial load expressed in velocity and a slope (Supplemental Table 2.EFG, Supplemental Digital Content 2, http://links.lww.com/PHM/B450). From these twelve studies, six studies made increments with velocity (Supplemental Table 2.E, Supplemental Digital Content 2, http://links.lww.com/PHM/B450^48,58,59,74–76^), five with slope (Supplemental Table 2.F, Supplemental Digital Content 2, http://links.lww.com/PHM/B450^16,37,50–52^) and one with velocity and slope (Supplemental Table 2.G, Supplemental Digital Content 2, http://links.lww.com/PHM/B450^1^). Two protocols expressed both their initial load and increments in push frequency and resistance (Supplemental Table 2.H, Supplemental Digital Content 2, http://links.lww.com/PHM/B450^38,68^). The initial load and/or increments were individually determined in 22 studies and based on (a combination of) a predicted test duration of ± 10 min (range: 6-14 min)^26,27,37,42,43,47,48,50–52,65,66,70^, another test on the same day^1,42,47,50–52,70,71^, previous visits to the lab^26,27,43,44,46,48,66,77^, participant characteristics^47,48,64,77^ or previous literature.^68,74,76^

The vast majority adopted a continuous protocol, three studies a discontinuous protocol.^30,73,76^ Step time was most often 1 min (18 studies, Supplemental Table 2, Supplemental Digital Content 2, http://links.lww.com/PHM/B450) and ranged between 15 s^35^ and 4 min.^30^ Test duration was reported in 19 studies and ranged from six to just over 18 min (Supplemental Table 2, Supplemental Digital Content 2, http://links.lww.com/PHM/B450).

***Outcomes***

*Wheelchair-specific anaerobic exercise capacity*

POmean was reported in eight studies and was calculated over the entire duration of the test (Supplemental Table 1, Supplemental Digital Content 2, http://links.lww.com/PHM/B450). POmax was reported in five studies and calculated in different ways. Four studies defined it as a one sample peak value^62,63,66,67^ and one study defined it as a 1s maximum.^66^ P5 was reported four times as the highest mean power over a successive 5 s interval. The RF was reported twice, where different definitions were used: (P5start – P5end)/P5start * 100% and (P5max – P5min)/P5max * 100%.^40,67^ Vmean and Vmax were reported in six and three studies, respectively. Some studies reported extra power-related outcomes, e.g. POmean/push or peak power after three cycles.^39,62,63,65^

*Wheelchair-specific aerobic exercise capacity*

POpeak was reported in nine studies and was defined as the average power output over the final stage of that protocol, which varied from 30 s to four min in studies that included the POpeak (Supplemental Table 2, Supplemental Digital Content 2, http://links.lww.com/PHM/B450). VO_2_peak was reported in all but one study^59^, 30 times as an absolute value [l/min] and 29 times relative to body mass [ml/min/kg] (22 studies reported both). It was defined as the average over a range from 10 to 60 s. HRpeak was reported in 32 studies, which varied from the highest one sample peak value to a 60 s average. RERpeak was reported in 17 studies with a similar time interval as the VO_2_peak. The RPE was reported in four studies: the 6-20 Borg scale was used three times^37,48,74^, the 1-10 once.^71^

**Discussion**

The aim of this review was to scope the literature on the different protocols used for lab-based wheelchair-specific anaerobic and aerobic exercise capacity testing in handrim wheelchair athletes. As expected, wheelchair athletes included in these studies form a heterogeneous group in terms of age, sport, disability, TSI etc. Additionally, testing of this group was conducted with a large variety in equipment and protocol design that inherently may influence the outcomes. Also, the reported outcomes of the tests were not identical among studies, even if they reported the same variable name. The heterogeneity of the test population, the diversity in equipment, the variety in used test protocols, and the different reported outcomes hamper comparison of study-material and drawing conclusions about the influence of specific protocol characteristics. In order to improve comparison and interpretation in future research, awareness among researchers needs to be improved on the smaller and larger effects of all those sources of variation among studies presented. Effects of variability of participant characteristics, equipment, protocol design and outcome definitions on the actual comparability of the outcomes will therefore be critically discussed below. Subsequently, the second aim was to synthesize the reviewed literature into a standardized, yet individualized protocol for future use in handrim wheelchair athletes. The suggested test protocol and reporting guidelines for future research, with a fictional example in the Supplementary Appendix 2 (Supplemental Digital Content 3, http://links.lww.com/PHM/B451), will be presented.

***Participant characteristics***

Participant characteristics were not consistently reported among the included studies. TSI, experience in sport and TH were missing in half of the studies. To get a total overview of the studied population and to be able to compare with other studies, these characteristics should be mentioned.^40,47,78^ We strongly advise to report all relevant participant characteristics in future studies, i.e. sex, age, body mass, height, sport, competition level, impairment, classification, time since injury, sport experience and training hours per week, while they affect peak performance outcome measures and simply help interpretation of the presented data.

***Equipment***

The specifications and measurement modalities differ between treadmills, integrated- and roller ergometers, but also within one kind of device differences are present which complicates comparison of study outcomes between apparatus.^22^ Treadmills offer realistic over-ground wheelchair propulsion in terms of friction, inertia and coasting behavior, requiring steering , but do not allow anaerobic testing.^79^ In ergometers steering is not essential, but ‘on-screen’ feedback can be a critical task characteristic.^80^ Since the majority of the included studies used a treadmill, this may explain why fewer studies focused on anaerobic capacity, compared to aerobic capacity (11 vs. 40 studies). Both capacities are, at least to some extent, important in wheelchair court and racing sports and can both be measured on an integrated or roller ergometer.

The majority of the included studies tested the athletes in their own competition wheelchair, which increases the sport-specific validity of the test.^81^ This notion is stressed in studies that evaluated small design changes (e.g. seat height or rim diameter) in the wheelchair-user interface on their effects in metabolic cost, mechanical efficiency and propulsion technique.^82–84^ Any changes in the individualized sports wheelchair may affect peak performance outcomes. Since wheelchair athletes have highly individualized wheelchairs, we advise to do performance tests in their own competition wheelchair, and thus on a wheelchair roller ergometer. To better understand results over time, it is highly recommended to report the wheelchair settings in as much detail as possible at every test moment (e.g. seat positioning, rear wheel camber, wheel/hand-rim size, mass).^81^

***Protocol design***

*Wheelchair-specific anaerobic exercise capacity*

Sprint and Wingate protocols differ in the set resistance and the reached mean velocity. Resistance was generally lower and mean velocity higher in sprint tests. The power output outcomes (POmean, POmax, P5) for the Wingate protocols seemed to be lower in comparison with the sprint protocols which is the opposite of the reasoning provided by the studies that used a Wingate protocol.^40,62,65,68^ Due to the lower velocity, as a consequence of the higher test resistance, the athlete would not be exposed to coordination problems of the upper body and potentially would reach higher anaerobic power output values.^10^ This contradictory result has a few potential explanations. Studies included different groups of athletes, used varying equipment with several protocols and different outcome measures were reported. For example, the highest POmean and POmax were found in a 5 s sprint protocol, with a flying start and with a tennis racket, that included wheelchair tennis athletes.^63^ The lowest POmean and P5 were found in a 30 s Wingate protocol, from stationary start, that included wheelchair track athletes from the lowest classification group.^40^ P5 was not reported in the first study, POmax not in the second. The same fixed wheelchair ergometer was used, while differently adjusted to the participant (daily living vs. individual competition wheelchair). It is impossible to derive the influence of a sprint or Wingate protocol on test outcomes, because of numerous differences in included participants, equipment, protocol design and reported outcomes. This underlines the need for better standardization.

Conclusions about the most appropriate anaerobic test are thus difficult to draw from this review due to the many differences between studies. If the aim is to measure the anaerobic capacity, rather than to mimic a field-test sprint, we do suggest a Wingate test with an increased resistance to avoid coordination problems of the upper body and reach a higher anaerobic power.^40,62,65,68^ To make sure the anaerobic energy system is maximally triggered, we opt for a duration of 30 s.^85^ A stationarity start is preferable over a flying start because it is the most standardized.

Studies that included a Wingate test set a resistance in order to keep the wheel velocity below 3 m/s.^40,62,65^ The resistance was set individually by the researchers based on participant characteristics. However, the exact ‘how-to’ was unclear, which hampers reproducibility. Studies in the field of rehabilitation that performed a Wingate test had the same aim, to stay below 3 m/s, but had a more standardized way of calculating the individual resistance.^86–90^ It was calculated based on an estimated power output (through a previous test) and a mean wheel velocity of 2 m/s. Wingate tests in this review have been performed in either fixed wheelchairs^40,62,86–90^ or in a rugby wheelchair.^65^ Racing wheelchairs have much smaller hand rim diameters which allows for higher wheel velocities with the same linear hand velocity (at the cost of a higher resistance).^84^ Thus, instead of aiming for a mean wheel velocity of 2 m/s, we suggest to rather standardize for a mean linear hand velocity of 2 m/s.^10^

*Wheelchair-specific aerobic exercise capacity*

The seven identified protocols differ in type of initial load and increment type. Similar to the anaerobic protocol, but with even more studies, comparisons between protocol types are difficult to make because of the heterogeneity of wheelchair athletes, the use of different equipment, different protocol design and the lack of identical outcome measures.

The majority of the studies made increments in (solely or partly) velocity (21 studies, Supplemental Table 2.ACE, Supplemental Digital Content 2, http://links.lww.com/PHM/B450). This results in high propulsion velocities which may lead to coordination problems of the upper extremities, rather than to their maximal aerobic capacity.^10^ To minimize the influence of possible high-speed coordination problems, a protocol with increments in resisting force is preferred. This can be done either by increasing the slope of a treadmill, by using a pulley system and adding more weight to the pulley on a treadmill, or by increasing the resistance of an ergometer. However, sole increments in slope will results in high slopes and lifting of the front wheels of the wheelchair can occur.^33^ By using increments in resisting force, propulsion velocity remains constant, and can be set to a comfortable speed matched to the participant’s ability, also done by multiple studies included in this review.^44,47,50–52,71^

The vast majority adopted a continuous protocol which is suggested to give similar results as a discontinuous protocol and is less time-consuming.^91^ One-third of the included studies explained that the magnitude of the initial load and increment were chosen in order to aim for a certain test duration (range: 6-14 min).^26,27,37,42,43,47,48,50–52,66^

Protocols with a step length of 3 min are often used to reach a steady-state in each step which is sometimes preferred by researchers to determine the submaximal mechanical efficiency and ventilatory thresholds, where the latter can be used for training purposes.^92^ However, a study that investigated the peak physiological responses and the determination of ventilatory thresholds between a 3 min, 1 min and RAMP protocol, using arm-crank ergometry, showed no differences in the identification of ventilatory thresholds.^20^ Also, a higher POpeak was found for the shorter protocols.^20^ It needs to be noted that no studies were included in this review that adopted a RAMP protocol with a linear increase in workload. Further research is needed to investigate the utility in wheelchair exercise tests of such a RAMP protocol. For now, a continuous protocol with an aimed duration between 8-12 min^19^ and steps of 1 min is recommended.

***Outcomes***

For the anaerobic test we suggest to adhere to the original outcomes of the Wingate test^69^, which are also the most often used among the included studies. This contains the POmean, POmax and P5 [W], which are calculated as the average power output over the total test duration, the one sample highest value and the highest mean power over successive 5 s intervals, respectively. RF as (P5start – P5end) / (P5start) * 100 %. Lastly, Vmean and Vmax [m/s].

For the aerobic test we suggest to use the VO_2_peak as a 30 s average [l/min and ml/min/kg]. The POpeak [W and W/kg] of the last completed step plus every 30 s in the non-completed step is taken into account by adding for each 30 s: 1 / (step length / 30 s) times the PO increment in the non-competed step.^93^ Secondary criteria are often used to assess the validity of the VO_2_peak (VO_2_ plateau (≤ 2 ml/min/kg change) over the last two stages, HRpeak > 95% of age-predicted maximum and RERpeak > 1.10).^18^ HRpeak is defined as the highest peak value and RERpeak in the same time interval as the VO_2_peak. Because the HR regulation might be affected in individuals with a SCI > T6, we advise to additionally use an RPE above 8 or 18 as a secondary criteria, depending on the scale.^18^ Lab-tests often adopt the 6-20 scale, field-tests 1-10.^94,95^ However, these can be converted to each other and thus interchangeably used.^96^

***Suggested guidelines for the standardization of (an)aerobic test protocols***

The resistance in the Wingate test and the increments in the GXT needs to be individually determined. Research found in this review did this mainly based on previous visits and/or participant characteristics.^26,37,40,42,43,47,50–52,62,65,66,70^ Yet, no general guidelines on why and how the specific resistances and increments were chosen were presented, hampering the reproducibility by other researchers.

A number of rehabilitation studies^86–90^ have adopted a standardized, yet individualized protocol that uses the relation between isometric force-anaerobic and aerobic power as a predictor for the most suited resistance.^97^ Isometric force can be measured in a standardized way and performed by each individual with the same settings.^86–89,98^ The participants exerts maximal force for 5 s on the top of the blocked hand-rims (ergometer or alternative set up).^86,87^ The before-mentioned associations are more specifically between the highest 3 s isometric user force, the mean power over a 30 s Wingate test (POmean) and the peak power in an GXT (POpeak).^97^ Thus, if the isometric force is measured, POmean can be predicted and with a mean hand velocity of 2 m/s the individual resistance of the Wingate test can be calculated. Similar, POpeak can be predicted from POmean and resistance increments can be individually scaled to achieve a duration of 8-12 min in the GXT. All calculations are presented in the Supplementary Appendix 2 (Supplemental Digital Content 3, http://links.lww.com/PHM/B451).

***Future research***

In order to draw conclusions about the influence of protocol characteristics on both the wheelchair-specific anaerobic and aerobic exercise capacity outcomes, aspects of protocol design should be investigated in isolation. The same group of individuals should be tested twice with only one change in the protocol design (e.g. stationary vs flying start in the anaerobic test). Such studies are currently lacking in the wheeled adapted sports domain.

In addition, the common features of equipment and test protocols discussed in this review were united in a proposed protocol for future standardisation, which is summarized in the Supplementary Appendix 2 (Supplemental Digital Content 3, http://links.lww.com/PHM/B451). A roller ergometer allows anaerobic exercise tests and the use of the athlete’s individualized competition wheelchair.^22^ To build the protocol for (an)aerobic exercise tests, associations between the isometric force-anaerobic and aerobic power can be used to predict outcomes, and accordingly, protocols can be individually scaled.^97^ Together with guidelines regarding participant characteristics and test outcomes, it becomes possible to compare among the various groups of wheelchair athletes and allow an easier interpretation of test outcomes.

These associations between the isometric force-anaerobic power and aerobic power were developed among a group of handrim wheelchair dependent male individuals with a chronic spinal cord injury^97^ and the use of this protocol is still limited to few rehabilitation and experimental studies.^86–89^ Previous research in male wheelchair track athletes showed a similar association between the anaerobic and aerobic capacity what might indicate that these associations are somehow similar in wheelchair athletes.^47^ However, future research should evaluate the usability of this association and protocol in a group of wheelchair athletes. It remains questionable if the previously found associations allow scaling of the test protocols in all individuals or whether they need to be reconsidered for each sport and/or classification.

**Conclusion**

With this scoping review we provided a highly variable perspective on the athlete population, equipment, test protocols and reported outcomes of the wheelchair-specific exercise capacity in international literature. This variability in equipment (e.g. ADL-wheelchair or sports wheelchair), test protocols (e.g. step time aerobic test) and reported outcomes (e.g. POmean and/or P5 and/or POmax in the anaerobic test) makes it difficult to interpret the anaerobic and aerobic test outcomes and compare results among the heterogeneous group of wheelchair athletes (e.g. practiced sport, TH/week). Yet, the common features were united into a proposed protocol and reporting guidelines for future standardisation. These guidelines should at least be used to report all relevant participant characteristics, equipment specifications, protocol design and the same test outcomes. Preferable researchers should adopt the standardized, yet individualised protocol for wheelchair-specific anaerobic and aerobic exercise capacity testing. By having a protocol based on common principles, albeit scaled to the individual, athletes will be provided with inter- and intra-athlete comparable measurements of their handrim wheelchair-specific exercise capacity, to ultimately improve their sport performance.

**Acknowledgements**

This review was funded by ZonMw (Wheelpower, project number: 546003002).

**Conflict of Interest statement**

The authors declare that they have no conflict of interest.

**References**

1. Veeger HEJ, Yahmed MH, Van der Woude LHV, Charpentier P. Peak oxygen uptake and maximal power output of Olympic wheelchair-dependent athletes. *Med Sci Sports Exerc*. 1991;23(10):1201-1209. doi:10.1249/00005768-199110000-00015

2. Goosey-Tolfrey V. Supporting the paralympic athlete: focus on wheeled sports. *Disabil Rehabil*. 2010;32(26):2237-2243. doi:10.3109/09638288.2010.491577

3. Barbosa TM, Forte P, Estrela JE, Coelho E. Analysis of the Aerodynamics by Experimental Testing of an Elite Wheelchair Sprinter. *Procedia Eng*. 2016;147(2):2-6. doi:10.1016/j.proeng.2016.06.180

4. Hedrick B, Wang YT, Moeinzadeh M, Adrian M. Aerodynamic positioning and performance in wheelchair racing. *Adapt Phys Act Q*. 1990;7:41-51. doi:10.1123/apaq.7.1.41

5. Goosey-Tolfrey VL, Fowler NE, Campbell IG, Iwnicki SD. A kinetic analysis of trained wheelchair racers during two speeds of propulsion. *Med Eng Phys*. 2001;23(4):259-266. doi:10.1016/S1350-4533(00)00084-9

6. de Klerk R, Vegter RJK, Leving MT, de Groot S, Veeger DHEJ, van der Woude LH V. Determining and Controlling External Power Output During Regular Handrim Wheelchair Propulsion. *J Vis Exp*. 2020;(156). doi:10.3791/60492

7. Baumgart JK, Brurok B, Sandbakk Ø. Peak oxygen uptake in Paralympic sitting sports: A systematic literature review, meta- and pooled-data analysis. *PLoS One*. 2018;13(2):1-25. doi:10.1371/journal.pone.0192903

8. Van der Woude LHV. *Sportwetenschappelijk Onderzoek: Rolstoelsport*.; 1996.

9. Theisen D, Francaux M, Fayt A, Sturbois X. A new procedure to determine external power output during handrim wheelchair propulsion on a roller ergometer: a reliability study. *Int J Sports Med*. 1996;17(8):564-571. doi:10.1055/s-2007-972896

10. Veeger HEJ, van der Woude LHV, Rozendal RH. Within cycle characteristics of the wheelchair push in sprinting on a wheelchair ergometer. *Med Sci Sports Exerc*. 1991;23(2):264-271. doi:10.1249/00005768-199102000-00019

11. Riebe D, Ehrman JK, Liguori G, Magal M. *ACSM’s Guidelines for Exercise Testing and Prescription*.; 2018. doi:10.1017/CBO9781107415324.004

12. Driss T, Vandewalle H. The measurement of maximal (anaerobic) power output on a cycle ergometer: a critical review. *Biomed Res Int*. Published online 2013. doi:10.1155/2013/589361

13. Haisma JA, Van der Woude LHV, Stam HJ, Bergen MP, Sluis TAR, Bussmann JBJ. Physical capacity in wheelchair-dependent persons with a spinal cord injury: A critical review of the literature. *Spinal Cord*. 2006;44:642-652. doi:10.1038/sj.sc.3101915

14. Van den Berg R, De Groot S, Swart KMA, Van der Woude LHV. Physical capacity after 7 weeks of low-intensity wheelchair training. *Disabil Rehabil*. 2010;32(26):2244-2252. doi:10.3109/09638288.2010.535688

15. Stewart MW, Melton-Rogers SL, Morrison S, Figoni SF. The measurement properties of fitness measures and health status for persons with spinal cord injuries. *Arch Phys Med Rehabil*. 2000;81(4):394-400. doi:10.1053/mr.2000.4417

16. Knechtle B, Köpfli W. Treadmill exercise testing with increasing inclination as exercise protocol for wheelchair athletes. *Spinal Cord*. 2001;39(12):633-636. doi:10.1038/sj.sc.3101229

17. Powers S, Howley E. *Exercise Physiology: Theory and Application to Fitness and Performances*.; 1995. doi:10.1249/00005768-199503000-00027

18. Goosey-tolfrey VL. *Bases Physiological Testing Guidelines : The Disabled Athlete*.; 2008.

19. Buchfuhrer MJ, Hansen JE, Robinson TE, Sue DY, Wasserman K, Whipp BJ. Optimizing the exercise protocol for cardiopulmonary assessment. *J Appl Physiol Respir Environ Exerc Physiol*. 1983;55(5):1558-1564. doi:10.1152/jappl.1983.55.5.1558

20. Kouwijzer I, Valize M, Valent LJM, Grandjean Perrenod Comtesse P, Van der Woude LHV, De Groot S. The influence of protocol design on the identification of ventilatory thresholds and the attainment of peak physiological responses during synchronous arm crank ergometry in able-bodied participants. *Eur J Appl Physiol*. 2019;119(10):2275-2286. doi:10.1007/s00421-019-04211-9

21. Eerden S, Dekker R, Hettinga FJ. Maximal and submaximal aerobic tests for wheelchair-dependent persons with spinal cord injury: a systematic review to summarize and identify useful applications for clinical rehabilitation. *Disabil Rehabil*. 2018;40(5):497-521. doi:10.1080/09638288.2017.1287623 LK

22. De Klerk R, Vegter RJK, Goosey-Tolfrey VL, et al. Measuring handrim wheelchair propulsion in the lab: A critical analysis of stationary ergometers. *IEEE Rev Biomed Eng*. 2020;13:199-211. doi:10.1109/RBME.2019.2942763 LK

23. Tricco AC, Lillie E, Zarin W, et al. PRISMA extension for scoping reviews (PRISMA-ScR): Checklist and explanation. *Ann Intern Med*. 2018;169(7):467-473. doi:10.7326/M18-0850

24. Arksey H, O’Malley L. Scoping studies: Towards a methodological framework. *Int J Soc Res Methodol Theory Pract*. 2005;8(1):19-32. doi:10.1080/1364557032000119616

25. Peters MDJ, Godfrey CM, Khalil H, McInerney P, Parker D, Soares CB. Guidance for conducting systematic scoping reviews. *Int J Evid Based Healthc*. 2015;13(3):141-146. doi:10.1097/XEB.0000000000000050

26. Diaper NJ, Goosey-Tolfrey VL. A physiological case study of a paralympic wheelchair tennis player: Reflective practise. *J Sport Sci Med*. 2009;8(2):300-307.

27. Goosey-Tolfrey VL, Batterham AM, Tolfrey K. Scaling behavior of VO2peak in trained wheelchair athletes. *Med Sci Sports Exerc*. 2003;35(12):2106-2111. doi:10.1249/01.mss.0000099106.33943.8c

28. De Groot S, Bos F, Koopman J, Hoekstra AE, Vegter RJK. The effect of a novel square-profile hand rim on propulsion technique of wheelchair tennis players. *Appl Ergon*. 2018;71(October 2017):38-44. doi:10.1016/j.apergo.2018.04.001

29. Vinet A, Bernard PL, Poulain M, Varray A, Le Gallais D, Micallef JP. Validation of an incremental field test for the direct assessment of peak oxygen uptake in wheelchair-dependent athletes. *Spinal Cord*. 1996;34(5):288-293. doi:10.1038/sc.1996.52

30. Vanlandewijck YC, Spaepen AJ, Lysens RJ. Relationship between the level of physical impairment and sports performance in elite wheelchair basketball athletes. *Adapt Phys Act Q*. 1995;12(2):139-150. doi:10.1123/apaq.12.2.139

31. Perret C, Labruyère R, Mueller G, Strupler M. Correlation of heart rate at lactate minimum and maximal lactate steady state in wheelchair-racing athletes. *Spinal Cord*. 2012;50(1):33-36. doi:10.1038/sc.2011.97

32. Gass GC, Camp EM. Physiological characteristics of trained Australian paraplegic and tetraplegic subjects. *Med Sci Sports*. 1979;11(3):256-259.

33. Gass GC, Camp EM. Effects of prolonged exercise on highly trained traumatic paraplegic men. *J Appl Physiol*. 1987;63(5):1846-1852. doi:10.1152/jappl.1987.63.5.1846

34. West CR, Leicht CA, Goosey-Tolfrey VL, Romer LM. Perspective: does laboratory-based maximal incremental exercise testing elicit maximum physiological responses in highly-trained athletes with cervical spinal cord injury? *Front Physiol*. 2016;6(419). doi:10.3389/fphys.2015.00419

35. Perez Tejero J, Coteron J, Rabadan M, Sampedro J. Field test validation for wheelchair basketball players’ aerobic capacity assessment. *Eur J Hum Mov*. 2018;40:136-148.

36. Morgulec-Adamowicz N, Kosmol A, Molik B, Yilla AB, Laskin JJ. Aerobic, anaerobic, and skill performance with regard to classification in wheelchair rugby athletes. *Res Q Exerc Sport*. 2011;82(1):61-69. doi:10.1080/02701367.2011.10599722

37. Paulson TAW, Goosey-Tolfrey VL, Lenton JP, Leicht CA, Bishop NC. Spinal cord injury level and the circulating cytokine response to strenuous exercise. *Med Sci Sports Exerc*. 2013;45(9):1649-1655. doi:10.1249/MSS.0b013e31828f9bbb

38. Coutts KD. Peak oxygen uptake of elite wheelchair athletes. *Adapt Phys Act Q*. 1990;7(1):62-66. doi:10.1123/apaq.7.1.62

39. Goosey-Tolfrey VL, Vegter RJK, Mason BS, et al. Sprint performance and propulsion asymmetries on an ergometer in trained high- and low-point wheelchair rugby players. *Scand J Med Sci Sport*. 2018;28(5):1586-1593. doi:10.1111/sms.13056

40. Van der Woude LHV, Bakker WH, Elkhuizen JW, Veeger HEJ, Gwinn T. Anaerobic work capacity in elite wheelchair athletes. *Am J Phys Med Rehabil*. 1997;76(5):355-365. doi:10.1097/00002060-199709000-00002

41. Bernard PL, Mercier J, Varray A, Prefaut C. Influence of lesion level on the cardioventilatory adaptations in paraplegic wheelchair athletes during muscular exercise. *Spinal Cord*. 2000;38(1):16-25. doi:10.1038/sj.sc.3100956

42. Soylu Ç, Yıldırım NÜ, Akalan C, Akınoğlu B, Kocahan T. The relationship between athletic performance and physiological characteristics in wheelchair basketball athletes. *Res Q Exerc Sport*. 2020;00(00):1-12. doi:10.1080/02701367.2020.1762834

43. Goosey-Tolfrey VL, Tolfrey K. The multi-stage fitness test as a predictor of endurance fitness in wheelchair athletes. *J Sports Sci*. 2008;26(5):511-517. doi:10.1080/02640410701624531

44. Campbell IG, Williams C, Lakomy HKA. Physiological responses of endurance-trained male wheelchair athletes to a 10-kilometer treadmill time trial. *Adapt Phys Act Q*. 2002;19(4):496-508. doi:10.1123/apaq.19.4.496

45. Molik B, Kosmol A, Morgulec-Adamowicz N, et al. Comparison of Aerobic Performance Testing Protocols in Elite Male Wheelchair Basketball Players. *J Hum Kinet*. 2017;60:243-254. doi:10.1515/hukin-2017-0140

46. Campbell IG, Williams C, Lakomy HKA. Physiological and metabolic responses of wheelchair athletes in different racing classes to prolonged exercise. *J Sports Sci*. 2004;22(5):449-456. doi:10.1080/02640410410001675298

47. Van der Woude LHV, Bouten C, Veeger HEJ, Gwinn T. Aerobic work capacity in elite wheelchair athletes: A cross-sectional analysis. *Am J Phys Med Rehabil*. 2002;81(4):261-271. doi:10.1097/00002060-200204000-00004 LK

48. Leicht CA, Griggs KE, Lavin J, Tolfrey K, Goosey-Tolfrey VL. Blood lactate and ventilatory thresholds in wheelchair athletes with tetraplegia and paraplegia. *Eur J Appl Physiol*. 2014;114(8):1635-1643. doi:10.1007/s00421-014-2886-x

49. Leicht CA, Paulson TAW, Goosey-Tolfrey VL, Bishop NC. Salivary alpha amylase not chromogranin A reflects sympathetic activity: exercise responses in elite male wheelchair athletes with or without cervical spinal cord injury. *Sport Med - open*. 2017;3(1):1. doi:10.1186/s40798-016-0068-6

50. Leicht CA, Bishop NC, Goosey-Tolfrey VL. Mucosal immune responses to treadmill exercise in elite wheelchair athletes. *Med Sci Sports Exerc*. 2011;43(8):1414-1421. doi:10.1249/MSS.0b013e31820ac959

51. Leicht CA, Bishop NC, Goosey-Tolfrey VL. Submaximal exercise responses in tetraplegic, paraplegic and non spinal cord injured elite wheelchair athletes. *Scand J Med Sci Sports*. 2012;22(6):729-736. doi:10.1111/j.1600-0838.2011.01328.x

52. Leicht CA, Tolfrey K, Lenton JP, Bishop NC, Goosey-Tolfrey VL. The verification phase and reliability of physiological parameters in peak testing of elite wheelchair athletes. *Eur J Appl Physiol*. 2013;113(2):337-345. doi:10.1007/s00421-012-2441-6

53. van Ingen Schenau GJ. Some fundamental aspects of the biomechanics of overground versus treadmill locomotion. *Med Sci Sports Exerc*. 1980;12(4):257-261.

54. Van der Woude LHV, De Groot D, Hollander AP, Van Ingen Schenau GJ, Rozendal RH. Wheelchair ergonomics and physiological testing of prototypes. *Ergonomics*. 1986;29(12):1561-1573. doi:10.1080/00140138608967269

55. Niesing R, Eijskoot F, Kranse R, et al. Computer-controlled wheelchair ergometer. *Med Biol Eng Comput*. 1990;28:329-338.

56. Faupin A, Gorce P, Thevenon A. A wheelchair ergometer adaptable to the rear-wheel camber. *Int J Ind Ergon*. 2008;38(7-8):601-607. doi:10.1016/j.ergon.2008.01.008

57. Faupin A, Borel B, Meyer C, Gorce P, Watelain E. Effects of synchronous versus asynchronous mode of propulsion on wheelchair basketball sprinting. *Disabil Rehabil Assist Technol*. 2013;8(6):496-501. doi:10.3109/17483107.2012.756947

58. De Lira CAB, Vancini RL, Minozzo FC, et al. Relationship between aerobic and anaerobic parameters and functional classification in wheelchair basketball players. *Scand J Med Sci Sport*. 2010;20(4):638-643. doi:10.1111/j.1600-0838.2009.00934.x

59. Tropp H, Samuelsson K, Jorfeldt L. Power output for wheelchair driving on a treadmill compared with arm crank ergometry. *Br J Sports Med*. 1997;31(1):41-44. doi:10.1136/bjsm.31.1.41

60. Gass GC, Camp EM. Physiological characteristics of trained australian paraplegic and tetraplegic subjects. *Med Sci Sports Exerc*. 1979;11(3):256-259. doi:10.1249/00005768-197901130-00006

61. Rotstein A, Sagiv M, Ben-Sira D, et al. Aerobic capacity and anaerobic threshold of wheelchair basketball players. *Paraplegia*. 1994;32(3):196-201. doi:10.1038/sc.1994.36

62. Roeleveld K, Lute E, Veeger HEJ, Gwinn T, Van der Woude LHV. Power output and technique of wheelchair athletes. *Adapt Phys Act Q*. 1994;11(1):71-85. doi:10.1123/apaq.11.1.71 LK

63. De Groot S, Bos F, Koopman J, Hoekstra AE, Vegter RJK. Effect of holding a racket on propulsion technique of wheelchair tennis players. *Scand J Med Sci Sports*. 2017;27(9):918-924. doi:10.1111/sms.12701

64. Cooper RA, O’Connor TJ, Robertson RN, Langbein WE, Baldini FD. An investigation of the exercise capacity of the wheelchair sports USA team. *Assist Technol*. 1999;11(1):34-42. doi:10.1080/10400435.1999.10131983

65. Marcolin G, Petrone N, Benazzato M, et al. Personalized tests in paralympic athletes: Aerobic and anaerobic performance profile of elite wheelchair rugby players. *J Pers Med*. 2020;10(3):1-10. doi:10.3390/jpm10030118 LK

66. Goosey-Tolfrey VL. Physiological profiles of elite wheelchair basketball players in preparation for the 2000 Paralympic Games. *Adapt Phys Act Q*. 2005;22(1):57-66. doi:10.1123/apaq.22.1.57

67. Hutzler Y, Grunze M, Kaiser R. Physiological and dynamic responses to maximal velocity wheelchair ergometry. *Adapt Phys Act Q*. 1995;12(4):344-361. doi:10.1123/apaq.12.4.344

68. Coutts KD, Stogryn JL. Aerobic and anaerobic power of canadian wheelchair track athletes. *Med Sci Sports Exerc*. 1987;19(1):62-65. doi:10.1249/00005768-198702000-00013

69. Bar-Or O. The Wingate Anaerobic Test An Update on Methodology, Reliability and Validity. *Sport Med An Int J Appl Med Sci Sport Exerc*. 1987;4(6):381-394. doi:10.2165/00007256-198704060-00001

70. Paulson TAW, Bishop NC, Leicht CA, Goosey-Tolfrey VL. Perceived exertion as a tool to self-regulate exercise in individuals with tetraplegia. *Eur J Appl Physiol*. 2013;113(1):201-209. doi:10.1007/s00421-012-2426-5

71. West CR, Goosey-Tolfrey VL, Campbell IG, Romer LM. Effect of abdominal binding on respiratory mechanics during exercise in athletes with cervical spinal cord injury. *J Appl Physiol*. 2014;117(1):36-45. doi:10.1152/japplphysiol.00218.2014

72. Cooper RA, Horvath SM, Bedi JF, Drechsler-Parks DM, Williams RE. Maximal exercise response of paraplegic wheelchair road racers. *Paraplegia*. 1992;30(8):573-581. doi:10.1038/sc.1992.117

73. Schmid A, Huonker M, Stober P, et al. Physical performance and cardiovascular and metabolic adaptation of elite female wheelchair basketball players in wheelchair ergometry and in competition. *Am J Phys Med Rehabil*. 1998;77(6):527-533. doi:10.1097/00002060-199811000-00015

74. Perret C, Labruyère R, Mueller G, Strupler M. Correlation of heart rate at lactate minimum and maximal lactate steady state in wheelchair-racing athletes. *Spinal Cord*. 2012;50(1):33-36. doi:10.1038/sc.2011.97

75. Knechtle B, Müller G, Willmann F, Eser P, Knecht H. Fat oxidation at different intensities in wheelchair racing. *Spinal Cord*. 2004;42(1):24-28. doi:10.1038/sj.sc.3101548

76. Otto AK, Reer R, Holtfreter B, Riepenhof H, Schröder J. Physiological responses at the anaerobic threshold and at peak performance during arm crank ergometer diagnostics compared to wheelchair propulsion on a treadmill in elite wheelchair basketball players. *Sport Orthop Traumatol*. 2019;35(1):49-55. doi:10.1016/j.orthtr.2019.01.009

77. West CR, Leicht CA, Goosey-Tolfrey VL, Romer LM. Perspective: does laboratory-based maximal incremental exercise testing elicit maximum physiological responses in highly-trained athletes with cervical spinal cord injury? *Front Physiol*. 2016;6:1-6. doi:10.3389/fphys.2015.00419

78. L.H.V. V der W, W.H. B, J.W. E, et al. Propulsion technique and anaerobic work capacity in elite wheelchair athletes: Cross-sectional analysis. *Am J Phys Med Rehabil*. 1998;77(3):222-234. doi:10.1097/00002060-199805000-00007

79. Hutzler Y. Anaerobic fitness testing of wheelchair users. *Sport Med*. 1998;25(2):101-113. doi:10.2165/00007256-199825020-00003

80. Klerk R De, Vegter RJK, Veeger HEJ, Woude LHV Van Der. Technical note : a novel servo-driven dual-roller handrim wheelchair ergometer. *IEEE Trans Neural Syst Rehabil Eng*. 2020;28(4):1-9.

81. Mason BS, Van Der Woude LHV, Goosey-Tolfrey VL. The ergonomics of wheelchair configuration for optimal performance in the wheelchair court sports. *Sport Med*. 2013;43(1):23-38. doi:10.1007/s40279-012-0005-x

82. Van Der Slikke RMA, De Witte AMH, Berger MAM, Bregman DJJ, Veeger DJHEJ. Wheelchair mobility performance enhancement by changing wheelchair properties: What is the effect of grip, seat height, and mass? *Int J Sports Physiol Perform*. 2018;13(8):1050-1058. doi:10.1123/ijspp.2017-0641

83. Van der Woude LHV, Veeger HEJ, Rozendal RH, Sargeant TJ. Seat height in handrim wheelchair propulsion. *J Rehabil Res Dev*. 1989;26(4):31-50.

84. Van der Woude LH, Veeger HE, Rozendal RH, Van Ingen Schenau GJ, Rooth F, Van Nierop P. Wheelchair racing: effects of rim diameter and speed on physiology and technique. *Med Sci Sports Exerc*. 1988;20(5):492-500.

85. Zajac A, Jarzabek R, Waskiewicz Z. The diagnostic value of the 10− and 30-second wingate test for competitive athletes. *J Strength Cond Res*. 1999;13(1):16-19. doi:10.1519/00124278-199902000-00003

86. Van der Scheer JW, De Groot S, Vegter RJK, Veeger HEJ, Van der Woude LHV. Can a 15m-overground wheelchair sprint be used to assess wheelchair-specific anaerobic work capacity? *Med Eng Phys*. 2014;36(4):432-438. doi:10.1016/j.medengphy.2014.01.003

87. Van der Woude LHV, Van Croonenborg JJ, Wolff I, Dallmeijer AJ, Hollander AP. Physical work capacity after seven weeks of wheelchair training: effect of intensity in able-bodied subjects. *Med Sci Sports Exerc*. 1999;31(2):331-341. doi:10.1097/00005768-199902000-00018

88. Dallmeijer AJ, Hopman MT, van As HH, Van der Woude LHV. Physical capacity and physical strain in persons with tetraplegia; the role of sport activity. *Spinal Cord*. 1996;34(12):729-735. doi:10.1038/sc.1996.133

89. Dallmeijer AJ, Hopman MTE, Angenot ELD, Van der Woude LHV. Effect of training on physical capacity and physical strain in persons with tetraplegia. *Scand J Rehabil Med*. 1997;29(3):181-186.

90. Van der Scheer JW, De Groot S, Postema K, Veeger HEJ, Van der Woude LHV. Design of a randomized-controlled trial on low-intensity aerobic wheelchair exercise for inactive persons with chronic spinal cord injury. *Disabil Rehabil*. 2013;35(13):1119-1126. doi:10.3109/09638288.2012.709301

91. Rasche W, Janssen TWJ, Van Oers CAJM, Hollander AP, Van der Woude LHV. Responses of subjects with spinal cord injuries to maximal wheelchair exercise: comparison of discontinuous and continuous protocols. *Eur J Appl Physiol Occup Physiol*. 1993;66(4):328-331. doi:10.1007/BF00237777

92. Boone J, Bourgois J. The oxygen uptake response to incremental ramp exercise: Methodogical and physiological issues. *Sport Med*. 2012;42(6):511-526. doi:10.2165/11599690-000000000-00000

93. Kuipers H, Verstappen FTJ, Keizer HA, Geurten P, van Kranenburg G. Variability of aerobic performance in the laboratory and its physiologic correlates. *Int J Sports Med*. 1985;6(4):197-201. doi:10.1055/s-2008-1025839

94. Foster C, Florhaug JA, Franklin J, et al. A New Approach to Monitoring Exercise Training. *J Strength Cond Res*. 2001;15(1):109-115. doi:10.1519/00124278-200102000-00019

95. Haddad M, Stylianides G, Djaoui L, Dellal A, Chamari K. Session-RPE method for training load monitoring: Validity, ecological usefulness, and influencing factors. *Front Neurosci*. 2017;11(612). doi:10.3389/fnins.2017.00612

96. Hutchinson MJ, Kouwijzer I, de Groot S, Goosey-Tolfrey VL. Comparison of two Borg exertion scales for monitoring exercise intensity in able-bodied participants, and those with paraplegia and tetraplegia. *Spinal Cord*. Published online 2021. doi:10.1038/s41393-021-00642-4

97. Janssen TWJ, Van Oers CAJM, Hollander AP, Veeger HEJ, Van der Woude LHV. Isometric strength sprint power and anaerobic power in individuals with a spinal cord injury. *Med Sci Sports Exerc*. 1993;25(7):863-870. doi:10.1249/00005768-199307000-00016

98. van der Scheer JW, de Groot S, Tepper M, et al. Wheelchair-specific fitness of inactive people with long-term spinalcord injury. *J Rehabil Med*. 2015;47(4):330-337. doi:10.2340/16501977-1934

99. Totosy de Zepetnek JO, Au JS, Hol AT, Eng JJ, MacDonald MJ. Predicting peak oxygen uptake from submaximal exercise after spinal cord injury. *Appl Physiol Nutr Metab*. 2016;41(7):775-781. doi:10.1139/apnm-2015-0670

100. Cooper RRA, Baldini FD, Boninger ML. Physiological responses to two wheelchair-racing exercise protocols. *Neurorehabil Neural Repair*. 2001;15(3):191-195. doi:10.1177/154596830101500306

101. Conconi F, Grazzi G, Casoni I, et al. The Conconi test: Methodology after 12 years of application. *Int J Sports Med*. 1996;17(7):509-519. doi:10.1055/s-2007-972887

102. Coutts KD, Rhodes EC, McKenzie DC. Maximal exercise responses of tetraplegics and paraplegics. *J Appl Physiol Respir Environ Exerc Physiol*. 1983;55(2):479-482. doi:10.1152/jappl.1983.55.2.479

Figure Legends:

**Figure 1** Conceptual framework of wheelchair-specific exercise capacity testing in the context of the wheelchair athlete’s (and team’s) performance. The performance consists most importantly of the wheeling and the physical performance and is influenced by many factors. These aspects can be measured together in the field as the wheelchair mobility performance, and together with the sport specific skills it determines the athlete (and team) performance. In the lab they can be measured as wheelchair-specific exercise capacity, which can be divided in three main components: strength, anaerobic capacity and aerobic capacity. The capacity should be assessed to eventually improve training strategies, monitor and compare athletes, which subsequently helps them to perform better in the field. This review focusses on the measurement of anaerobic and aerobic capacity in the lab-based environment

**Figure 2** Flow chart of the literature selection process
