# Supplementary material for: Toward a Standardized and Individualized Laboratory-Based Protocol for Wheelchair-Specific Exercise Capacity Testing in Wheelchair Athletes: A Scoping Review
Source: Am J Phys Med Rehabil. 2021 Dec 21;102(3):261–9. doi: 10.1097/PHM.0000000000001941 (PMC9940834; doi:10.1097/PHM.0000000000001941)
Supplement: Supplementary file 4 [file ajpmr-102-261-s004.docx]

Title page

**Title:** A novel approach to severe chronic neurogenic dysphagia using pharyngeal sensory electrical stimulation: a case report

Authors: Xue Zhang ^1^, Xiaolu Wang ^2^, Zulin Dou ^1,^*, Hongmei Wen ^1,^*

**Affiliations:**

1 Department of Rehabilitation Medicine, The Third Affiliated Hospital of Sun Yat-sen University, Guangzhou, 510630, China

2 Key Laboratory of Sensing Technology and Biomedical Instrument of Guangdong Province, School of Biomedical Engineering of Sun Yat-sen University, Guangzhou, 510006, China

***Correspondence:**

Hongmei Wen, Email: [wenhongm@mail.sysu.edu.cn](mailto:wenhongm@mail.sysu.edu.cn), Telephone and Fax numbers: +86-20-85256013 (H.W.)

Zulin Dou, Email: [douzul@163.com](mailto:douzul@163.com), Telephone and Fax numbers: +86-20-85252357 (Z.D.)

All correspondence should be addressed to：Department of Rehabilitation Medicine, The Third Affiliated Hospital, Sun Yat-sen University，Guangzhou 510630, China

**Declarations**

**Funding:** This research was funded by the National Natural Science Foundation of China (Grant Numbers 81972159 and 81672259), the Natural Science Foundation of Guangdong Province of China (Grant Numbers 2019A1515010388 and 2020A1515010881), and the Clinical Research Special Fund Project of the Third Affiliated Hospital of Sun Yat-sen University (Voyage Plan) (YHJH201909).

**Conflicts of Interest:** None declared.

**Ethics approval:** The research proposal was approved by the Ethics Committee of The Third Affiliated Hospital of Sun Yat-sen University (no. [2021]02-259-01).

**Informed consent:** The patient signed written informed consent for this study.

**Abstract**

The treatment options for severe chronic neurogenic dysphagia are limited. A patient, after resection of medulla oblongata hemangioblastoma, who failed to respond to seven months of traditional dysphagia rehabilitation therapy, was treated with prolonged pharyngeal sensory electrical stimulation for 39 sessions over 57 days. For the first time, this case report showed improvement in hypopharyngeal peak pressure (9.1 mmHg vs. 90.8 mmHg) using high-resolution manometry. Reduction in the penetration and aspiration scale, secretion, and residue of the vallecular and pyriform sinus were verified by videofluoroscopic swallowing study and flexible endoscopic evaluation of swallowing. The Functional Oral Intake Scale score increased from 1 to 6. No adverse event was observed. This case report presented a potential therapeutic protocol for severe chronic neurogenic dysphagia, which might be instructive for clinical practice.

**Keywords:** pharyngeal electrical stimulation; dysphagia; hemangioblastoma; hypopharyngeal peak pressure; hypopharyngeal contractility

Introduction

Hypopharyngeal weakness, insufficient laryngo-hyoid complex elevation, and upper esophageal sphincter (UES) dysfunction are great challenges in the rehabilitation of severe chronic neurogenic dysphagia. Severe chronic neurogenic dysphagia refers to those patients with severe dysphagia secondary to central nervous system disease for more than three months. Severe chronic neurogenic dysphagia is associated with increased pneumonia, malnutrition, mortality, and cost ^1-3^. Regarding hypopharyngeal weakness, no proven evidence for effective treatments exists yet ^4,5^.

Pharyngeal electrical stimulation (PES) is a novel neurostimulation tool for dysphagia ^6-9^. However, no studies investigated the effects of PES on pressures along the pharynx and upper esophagus, and no standardized recognized PES protocol for treating severe chronic neurogenic dysphagia exists. In addition, the localization of the PES electrodes is cumbersome. PES requires the catheter surface electrode to be accurately placed on the hypopharyngeal surface. The intubation approaches vary in different studies and include rough calculations according to the participant’s height, inserting a length of 15 ~ 16 cm through the nose, intubating under gastrointestinal X-ray or laryngoscopy, or placement 3 cm above the UES after high-resolution manometry ^6-10^.

Pharyngeal sensory electrical stimulation (PSES) is a modified neurostimulation tool. Compared with PES, PSES electrode placement is facilitated using electromyography (EMG). Regarding the stimulation parameters, the PES uses square wave, pulse width 0.2 ms, frequency 5 Hz, and 10 min per day, while PSES uses mixed triangular and square wave, pulse width 10 ms, frequency 5 Hz, and 10 min per day. It was proposed that wide pulse duration might enhance the evoked sensory volley to the central nervous system^11-13^ and reduce the fatigability of contractions^14^. Meanwhile, the triangular waveform and wide pulse duration could synergistically stimulate the denervated muscle^15,16^. The standard treatment period for PES is three consecutive days but lacks evidence for PSES. Here, PSES was applied to a severe chronic neurogenic dysphagia patient with hypopharyngeal weakness to investigate its safety and therapeutic effects.

This case conforms to all CARE guidelines and reports the required information accordingly (see CARE-checklist, Supplemental Digital Content 1, http://links.lww.com/PHM/B853).

Case presentation

A 31-year-old male patient with a history of recurrent headaches and dizziness four years before admission on November 14, 2020. The magnetic resonance imaging (MRI) showed hemangioblastoma in the dorsolateral medulla oblongata (Supplementary Figure 1A,1B, Supplemental Digital Content 3, http://links.lww.com/PHM/B855). The patient underwent hemangioblastoma removal on November 15, 2020. After surgery, repeated MRI showed no intracranial hemorrhage, hydrocephalus, and abnormal tissue (Supplementary Figure 1C, Supplemental Digital Content 3, http://links.lww.com/PHM/B855). But the patient had persistent symptoms of dysphagia due to lingual sensorimotor deficits, cricopharyngeal dysfunction, and poor swallowing coordination. Besides that, the patient suffered from orthostatic hypotension, dystaxia, and hypodynamia. Even though the patient underwent routine swallowing rehabilitation, including transcutaneous neuromuscular electrical stimulation (NMES) (20 min/day, 5 days/week), cold thermal stimulation (20 min/day, 5 days/week), tongue exercises (20 min/day, 5 days/week), and catheter balloon dilation (8 times/day, 5 days/week) for seven months, he still could not eat orally. Therefore, PSES was delivered as a supplemental treatment, while NMES and cold thermal stimulation were discontinued due to cost and unsatisfactory treatment results.

Before starting the PSES, high-resolution manometry (ManoScan 360, Ltd., Los Angeles, CA, USA) ^5,17^ showed the hypopharyngeal peak pressure decreased (9.1 mmHg), the residual pressure of the UES relaxation increased (210.1 mmHg) (Figure 1 top row, white arrow). Flexible endoscopic evaluation of swallowing (ATMOS; MedizinTechnik GmbH & Co. KG, Lenzkirch, Germany) showed right vocal cord paralysis, aspiration of saliva (Murray secretion severity rating scale = 4) ^18^, severe residue (Yale Pharyngeal Residue Severity Rating Scale) ^19^, and aspiration (penetration and aspiration scale = 7 ^20^; Figure 1 top row, red arrow), and an inability to eat orally (Functional Oral Intake Scale = 1). Videofluoroscopic swallowing study ^21^ (PLD8100C, gastrointestinal X-ray machine; Perlove, Zhuhai, China) showed that contrast agent could not enter the esophagus and aspiration, even when a catheter balloon placed in the pharynx aid swallowing (penetration and aspiration scale = 7; Modified barium swallowing Measurement Tool for Swallow Impairment ^22^ Pharyngoesophageal Segment Opening = 3; Figure 1 top row, green arrow), and significantly reduced laryngo-hyoid complex elevation (Modified barium swallowing Measurement Tool for Swallow Impairment, Hyoid Motion = 2^22^; Laryngeal Elevation = 2^22^). (More details refer to Table 1, Figure 1 top row) (see also Supplementary Materials, Supplemental Digital Content 2, http://links.lww.com/PHM/B854).

After receiving written informed consent from the patient, PSES was tried on May 21, 2021. The PSES device (ZIMMER, Neu-Ulm, Germany) contained a tube with two pairs of electrodes (the stimulus electrodes, placed at 8.8 cm apart from the reference electrodes, are also used to record the EMG signals), a control panel for regulating parameters, and a portable EMG device. The stimulus was a mixed waveform with a frequency of 5 Hz and a pulse width of 10 ms (Supplementary Figure 2A, Supplemental Digital Content 4, http://links.lww.com/PHM/B856). The first time placing the electrodes, a laryngoscope guided them into the pharyngeal cavity through the nose. The stimulus electrode ring was placed within 1 cm of the piriform sinus, and the reference ring was placed in the upper esophagus (Supplementary Figure 2B, Supplemental Digital Content 4, http://links.lww.com/PHM/B856). At this time, the pharyngeal EMG stably dropped below 20 µV, indicating good contact between the stimulation electrode and the pharyngeal mucosa (Supplementary Figure 2C, Supplemental Digital Content 4, http://links.lww.com/PHM/B856). The depth of the stimulation electrode was 15 cm from the nostril to the piriform sinus. For subsequent electrodes placement, the tube was inserted from the same nostril guided by the EMG without the laryngoscope. When the EMG signal decreased for the first time and stabilized below 20 µV, the location of the tube was fixed. When the patient could swallow, the EMG value increased above 20 µV during swallowing (Supplementary Figure 2D, Supplemental Digital Content 4, http://links.lww.com/PHM/B856).

After the catheter position was fixed, the current intensity (CI) was detected. The CI was gradually increased from 0.5 mA. The perception threshold (PT) was the lowest CI at which the patient could feel the stimulation. The maximum tolerance threshold (MTT) was the CI at which the patient felt pain and discomfort and didn’t wish to increase further. The stimulus CI was calculated as $PT+\left( 0.75\times\left[ MTT-PT \right] \right)$. The stimulation duration was 10 min/day. PSES was conducted every day, except for weekends, holidays, or the patient felt uncomfortable. 200ml/time oral intake was set as the endpoint of the PSES in consideration of nutrient requirements and potential swallowing function improvement. A qualified physiatrist performed PSES in the laryngoscopy examining room and disinfected it after each use. When the patient could orally take porridge 200 mL/time on July 16, 2021, PSES was discontinued. After the end of PSES, routine rehabilitations, including tongue exercises (20 min/day, 5 days/week), catheter balloon dilation (8 times/day, 5 days/week), and therapeutic feeding (thickened liquids with chin tuck, 30 min/day, 5 days/week), were continued until hospital discharge to further ameliorate dysphagia caused by poor swallowing compliance and coordination, lingual weakness, and UES dysfunction. 10min/day of PSES was applied in 39 sessions over 57 days. The stimulation intensities varied across sessions (Supplementary Figure 2E, Supplemental Digital Content 4, http://links.lww.com/PHM/B856). The stimulus CI decreased from 20 to 4.4 ± 1.2 mA. No adverse reactions occurred.

The swallowing function was re-evaluated after the last PSES. High-resolution manometry, videofluoroscopic swallowing study, and flexible endoscopic evaluation of swallowing were performed by the same licensed speech language pathologist and qualified physiatrist before and after treatment. The results indicated improved hypopharyngeal peak pressure (90.8 mmHg) (Figure 1 bottom row, white arrow), hypopharyngeal contraction duration, velopharyngeal peak pressure, velopharyngeal contraction duration, residual pressure of UES relaxation (90.4 mmHg), and duration of relaxation, vocal cord mobility, secretion (Murray secretion severity rating scale = 2), residue, aspiration (penetration and aspiration scale =1, Figure 1 bottom row, red arrow), UES dysfunction (Modified barium swallowing Measurement Tool for Swallow Impairment, Pharyngoesophageal Segment Opening = 1, Figure 1 bottom row, green arrow), laryngo-hyoid complex elevation (Modified barium swallowing Measurement Tool for Swallow Impairment, Hyoid Motion = 1, Laryngeal Elevation = 0). (More details refer to Table 1, Figure 1 bottom row)

Despite the cessation of PSES, the swallowing function continued to improve gradually with routine rehabilitation (Supplementary Figure 3, Supplemental Digital Content 5, http://links.lww.com/PHM/B857). The patient could take most types of food (Functional Oral Intake Scale = 6) orally 120 days after discharge without adverse events. The timeframe of the case from the onset of illness until follow-up was presented in Supplementary Figure 4 (Supplemental Digital Content 6, http://links.lww.com/PHM/B858).

Discussion

This case report showed the potential of PSES in the treatment of dysphagia for the first time. Hypopharyngeal peak pressure between tongue base and UES under high-resolution manometry was the best predictor of residue and aspiration ^2,3^. Increased hypopharyngeal peak pressure contributed mostly to the improvements in residual, penetration and aspiration scale, and overall swallowing function. Surprisingly, UES dysfunction was also improved, which might be associated with increased hypopharyngeal peak pressure that provides sufficient bolus driving force, and improved laryngo-hyoid complex elevation that provides sufficient up-forward movement ^2,23^. Thus, PSES might be a novel treatment for patients with UES dysfunction and hypopharyngeal weakness ^4,24^.

Moreover, despite the cessation of PSES, the swallowing function continued to improve gradually. The possible reason is that after PSES, the hypopharyngeal contractility was improved, allowing for oral intake. Through continuous oral intake therapy, the compliance and coordination of swallowing were reconstructed, and the neuroplasticity of the swallowing cortex was triggered. Thus, the swallowing function continued to improve and maintained.

PSES electrodes can be accurately located within 5 minutes with a laryngoscope and EMG device. Compared with the stimulation intensity of PES, which often exceeds 10 mA in patients ^6-8^, the stimulation intensity of PSES is relatively lower at 4.4 ± 1.2 mA, which may be due to the modified hybrid waveform and 10-ms pulse width of PSES. The gradually declined stimulation intensity might indicate restored pharyngeal sensation, which is associated with residue and aspiration, consistent with previous findings ^25^. The underlying mechanism of PSES might be similar to that of PES, which can modulate the organization and behavior of the swallowing network ^8-10^. Future studies are needed to investigate the mechanism of PSES.

The limitation is that this is a before-after case without randomized control. Nevertheless, the patient did not improve after seven months of routine therapy and began to recover gradually after PSES. Thus, the therapeutic effect might be mainly caused by the prolonged PSES, and self-recovery may contribute relatively minor. The PSES protocol in severe chronic hypopharyngeal weakness is likely longer than the standard protocol of PES. Further studies with large sample randomized controlled trials should be adopted to clarify the effect of PSES on severe chronic neurogenic dysphagia with poor hypopharyngeal contractility.

Conclusion

This case report explored the possible role of a novel therapeutic protocol for severe chronic neurogenic dysphagia with hypopharyngeal weakness, suggesting that PSES might be a potential therapeutic option for severe chronic neurogenic dysphagia with hypopharyngeal weakness. Further investigations with a large sample size and randomized control trials are needed to validate the effectiveness of PSES on severe dysphagic patients.

**References**

1. Rommel N, Hamdy S. Oropharyngeal dysphagia: manifestations and diagnosis. *Nat Rev Gastroenterol Hepatol*. Jan 2016;13(1):49-59. doi:10.1038/nrgastro.2015.199

2. Bayona HHG, Pizzorni N, Tack J, Goeleven A, Omari T, Rommel N. Accuracy of High-Resolution Pharyngeal Manometry Metrics for Predicting Aspiration and Residue in Oropharyngeal Dysphagia Patients with Poor Pharyngeal Contractility. *Dysphagia*. Feb 19 2022;doi:10.1007/s00455-022-10417-5

3. Takasaki KU, H.; Enatsu, K.; Tanaka, F.; Sakihama, N.; Kumagami, H; Takahashi, H. Investigation of pharyngeal swallowing function using high-resolutionmanometry. *Laryngoscope*. 2008;118:1729-1732.

4. Martino R, McCulloch T. Therapeutic intervention in oropharyngeal dysphagia. *Nat Rev Gastroenterol Hepatol*. Nov 2016;13(11):665-679. doi:10.1038/nrgastro.2016.127

5. McCulloch TM HM, Ciucci MR. High resolution manometry of pharyngeal swallow pressure events associated with head turn and chin tuck. *Ann Otol Rhinol Laryngol*. 2010;119(6):369-76. doi: https://doi.org/10.1177/000348941011900

6. Bath PM, Woodhouse LJ, Suntrup-Krueger S, et al. Pharyngeal electrical stimulation for neurogenic dysphagia following stroke, traumatic brain injury or other causes: Main results from the PHADER cohort study. *EClinicalMedicine*. Nov 2020;28:100608. doi:10.1016/j.eclinm.2020.100608

7. Dziewas R, Stellato R, van der Tweel I, et al. Pharyngeal electrical stimulation for early decannulation in tracheotomised patients with neurogenic dysphagia after stroke (PHAST-TRAC): a prospective, single-blinded, randomised trial. *The Lancet Neurology*. 2018;17(10):849-859. doi:10.1016/s1474-4422(18)30255-2

8. Jayasekeran V, Singh S, Tyrrell P, et al. Adjunctive functional pharyngeal electrical stimulation reverses swallowing disability after brain lesions. *Gastroenterology*. May 2010;138(5):1737-46. doi:10.1053/j.gastro.2010.01.052

9. Scutt P, Lee HS, Hamdy S, Bath PM. Pharyngeal Electrical Stimulation for Treatment of Poststroke Dysphagia: Individual Patient Data Meta-Analysis of Randomised Controlled Trials. *Stroke Res Treat*. 2015;2015:429053. doi:10.1155/2015/429053

10. Suntrup S, Teismann I, Wollbrink A, et al. Pharyngeal electrical stimulation can modulate swallowing in cortical processing and behavior - magnetoencephalographic evidence. *Neuroimage*. Jan 1 2015;104:117-24. doi:10.1016/j.neuroimage.2014.10.016

11. Bergquist AJ, Clair JM, Lagerquist O, Mang CS, Okuma Y, Collins DF. Neuromuscular electrical stimulation: implications of the electrically evoked sensory volley. *Eur J Appl Physiol*. Oct 2011;111(10):2409-26. doi:10.1007/s00421-011-2087-9

12. Donnelly C, Stegmüller J, Blazevich AJ, et al. Modulation of torque evoked by wide-pulse, high-frequency neuromuscular electrical stimulation and the potential implications for rehabilitation and training. *Sci Rep*. Mar 18 2021;11(1):6399. doi:10.1038/s41598-021-85645-0

13. Maffiuletti NA, Gondin J, Place N, Stevens-Lapsley J, Vivodtzev I, Minetto MA. Clinical Use of Neuromuscular Electrical Stimulation for Neuromuscular Rehabilitation: What Are We Overlooking? *Arch Phys Med Rehabil*. Apr 2018;99(4):806-812. doi:10.1016/j.apmr.2017.10.028

14. Barss TS, Ainsley EN, Claveria-Gonzalez FC, et al. Utilizing Physiological Principles of Motor Unit Recruitment to Reduce Fatigability of Electrically-Evoked Contractions: A Narrative Review. *Arch Phys Med Rehabil*. Apr 2018;99(4):779-791. doi:10.1016/j.apmr.2017.08.478

15. Pieber K, Herceg M, Paternostro-Sluga T, Schuhfried O. Optimizing stimulation parameters in functional electrical stimulation of denervated muscles: a cross-sectional study. *J Neuroeng Rehabil*. Jun 7 2015;12:51. doi:10.1186/s12984-015-0046-0

16. Chandrasekaran S, Davis J, Bersch I, Goldberg G, Gorgey AS. Electrical stimulation and denervated muscles after spinal cord injury. *Neural Regen Res*. Aug 2020;15(8):1397-1407. doi:10.4103/1673-5374.274326

17. Nativ-Zeltzer N, Logemann JA, Zecker SG, Kahrilas PJ. Pressure topography metrics for high-resolution pharyngeal-esophageal manofluorography-a normative study of younger and older adults. *Neurogastroenterol Motil*. May 2016;28(5):721-31. doi:10.1111/nmo.12769

18. Kuo CWACTHCCLCJ. Murray secretion scale and fiberoptic andoscopic evaluation of swallowing in predicting aspiration in dysphagic patients. *Eur Arch Otorhinolaryngol*. 2017;274(6):2513-2519. doi:10.1007/s00405-017-4522-y

19. Neubauer PD, Rademaker AW, Leder SB. The Yale Pharyngeal Residue Severity Rating Scale: An Anatomically Defined and Image-Based Tool. *Dysphagia*. Oct 2015;30(5):521-8. doi:10.1007/s00455-015-9631-4

20. John C. Rosenbek JAR, Ellen B. Roecker, Jame L. Coyle, and Jennifer L. Wood. A Penetration-aspiration Scale. *Dysphagia*. 1996;11:93-98. doi:10.1007/BF00417897

21. Costa MM. Videofluoroscopy: the gold standard exam for studying swallowing and its dysfunction. *Arq Gastroenterol*. Oct-Dec 2010;47(4):327-8. doi:10.1590/s0004-28032010000400001

22. Martin-Harris B, Brodsky MB, Michel Y, et al. MBS measurement tool for swallow impairment--MBSImp: establishing a standard. *Dysphagia*. Dec 2008;23(4):392-405. doi:10.1007/s00455-008-9185-9

23. Kendall KA, Leonard RJ. Videofluoroscopic upper esophageal sphincter function in elderly dysphagic patients. *Laryngoscope*. Feb 2002;112(2):332-7. doi:10.1097/00005537-200202000-00024

24. Xie M, Zeng P, Wan G, et al. The Effect of Combined Guidance of Botulinum Toxin Injection with Ultrasound, Catheter Balloon, and Electromyography on Neurogenic Cricopharyngeal Dysfunction: A Prospective Study. *Dysphagia*. Apr 29 2021;doi:10.1007/s00455-021-10310-7

25. Shapira-Galitz Y, Shoffel-Havakuk H, Halperin D, Lahav Y. Association Between Laryngeal Sensation, Pre-swallow Secretions and Pharyngeal Residue on Fiberoptic Endoscopic Examination of Swallowing. *Dysphagia*. Aug 2019;34(4):548-555. doi:10.1007/s00455-019-10001-4

**Figure Legends**

**Figure 1.** Auxiliary images before and after pharyngeal sensory electrical stimulation (PSES). High-resolution manometry showed an increase in the hypopharyngeal peak pressure (white arrow) from 9.1 mmHg to 90.8 mmHg after PSES. Flexible endoscopic evaluation of swallowing showed improvement in aspiration (red arrow) before and after PSES. Videofluoroscopic swallowing study showed improvement in the Pharyngoesophageal Segment Opening (green arrow) before and after PSES.

**Supplementary Figure 1**. (A, B) The magnetic resonance imaging (MRI) (Oct 15, 2020) showing hemangioblastoma of dorsolateral medulla oblongata (red arrow). (C) The MRI (Nov 12, 2020) showing no intracranial hemorrhage, hydrocephalus, and abnormal tissue.

**Supplementary Figure 2.** Parameters and configurations for PSES therapy. (A) Electrical stimulus parameters. (B) The stimulation electrode ring is placed within 1 cm of the piriform sinus. (C) The EMG value of the pharyngeal constrictor decreased and stabilized below 20 µV, indicating good contact between the stimulation ring electrode and the inferior pharyngeal mucosa. (D) When the patient’s swallowing function improved, the EMG value rapidly rose to more than 20 µV during swallowing. (E) Variation in the stimulation intensities.

**Supplementary Figure 3**. The volumes of orally taken food varied by time for the patient. The red arrow indicates the pharyngeal sensory electrical stimulation was stopped on Jul 16, 2021.

**Supplementary Figure 4**. The timeframe of the case from the onset of illness until follow-up.
